# Tracking the burden, distribution, and impact of Post-COVID conditions in diverse populations for children, adolescents, and adults (Track PCC): passive and active surveillance protocols

**DOI:** 10.1186/s12889-024-19772-4

**Published:** 2024-08-29

**Authors:** Resa M. Jones, Jennifer G. Andrews, Alexandra F. Dalton, Brian E. Dixon, Bari J. Dzomba, Shane I. Fernando, Kristen M. Pogreba-Brown, Miguel Reina Ortiz, Vinita Sharma, Nicole Simmons, Sharon H. Saydah, for the Track PCC Study Group, Joshua Slen, Lillian Smith, Joanna McComack, Mac McCullough, Brian Young, Megha Khatri Arora, Rebekah Epstein, Ralph Figueroa, Terry Mahotiere, Kathryn Miller, Lori Barrett, McKenna Dahlquist, Dolores Busch, Tracy Edinger, Pablo Garcia, Richard Gibson, Sara Hallvik, Emily Sim, Christian Flessner, Navina Forsythe, Maria Johnson, Ryan McLelland, Joseph Sorenson, Fatima Ayllon, Marina Oktapodas Feiler, Matthew Fukuhara, Aaron Mishkin, Thanh T. D. Phan, Mehdi Rajaeebaygi, Radhika Sinha, John Turella, Weiting Wang, Lucie Wiedefeld, Recai Yucel, Susan Robinson, Argelia Benavides, Kate Bessey, Shane Brady, Collin Catalfamo, Dametreea Carr, Clancey Collins, Felina Cordova-Marks, Kacey Ernst, Leslie Farland, Pamela Gracia-Filion, Scott Frost, Kelly Heslin, Elizabeth Jacobs, Priscilla Lauro, Velia Nuno, Sydney Pettygrove, Vern Pilling, Susan Robinson, Alexandra Shilen, Vignesh Subbian, Shaun Grannis, Katie Allen, Lauren Buelow, Aaron Buck, Tom Duszynski, William Fadel, Zamal Franks, Ashley Griffith, Laura J. Myers, John Price, Ashley Wiensch, Hiping Xu, Abe Agedew, Deja Edwards, Emily Koumas, Douglas Slaughter, Elizabeth Sullivan, Tracy Wyche, Jason Brinkley, Tana Brummer, Sameer Desale, Rebecca Devlin, Charles Harpole, Danielle Rentz Hunt, Zuha Jeddy, Brandon Poe, Steve Pickett, Erica Sewell, Brian Sokol, Karen Stein, Joseph Thomas

**Affiliations:** 1 Department of Epidemiology and Biostatistics, College of Public Health, Temple University, 1301 Cecil B. Moore Ave., Ritter Annex, 9th Floor, Rm 917, Philadelphia, PA 19122 USA; 2 Fox Chase Cancer Center, Temple University Health, Philadelphia, PA USA; 3 Department of Pediatrics, University of Arizona, Tucson, AZ USA; 4 Centers for Disease Control and Prevention, Atlanta, GA USA; 5 Department of Health Policy & Management, Fairbanks School of Public Health, Indiana University, Indianapolis, IN USA; 6 Center for Biomedical Informatics, Regenstrief Institute, Indianapolis, IN USA; 7 Department of Veterans Affairs, Center for Health Information and Communication, Health Services Research & Development Service, Indianapolis, IN USA; 8 Department of Health Services Administration and Policy, College of Public Health, Temple University, Philadelphia, PA USA; 9 Abt Global, Rockville, MD USA; 10 Department of Pediatrics and Women’s Health, University of North Texas Health Science Center, Fort Worth, TX USA; 11 Department of Epidemiology and Biostatistics, Zuckerman College of Public Health, University of Arizona, Tucson, AZ USA; 12 Department of Community and Global Health, Fairbanks School of Public Health, Indiana University, Indianapolis, IN USA

**Keywords:** Cohort study, Incidence, Post-COVID conditions, Prevalence, Public health, Surveillance

## Abstract

**Background:**

Track PCC includes five geographic surveillance sites to conduct standardized population-based surveillance to estimate and track Post-COVID Conditions (PCC) by age, sex, race/ethnicity, geographic area, severity of initial infection, and risk factors among persons with evidence of SARS-CoV-2 infection (based on the Council of State and Territorial Epidemiologist [CSTE] case definitions for confirmed cases or laboratory-confirmed evidence of infection).

**Methods:**

The study will estimate the incidence, prevalence, including temporal trends, and duration and severity of PCC symptoms, among children, adolescents, and adults. PCCs include a broad range of symptoms and conditions that continue or develop after acute SARS-CoV-2 infection or COVID-19 illness. Surveillance includes both passive and active components for diverse populations in Arizona, Indiana, and Utah as well as the Bronx Borough, NY, and part of Philadelphia County, PA. Passive surveillance will utilize electronic health records and health information exchanges within each site catchment area to longitudinally follow persons with COVID-19 to estimate PCC occurring at least 30 days after acute COVID-19 illness. Active surveillance will utilize self-report of PCCs from detailed surveys of persons ages 7 years and older with evidence of SARS-CoV-2 infection in the past 3 months. Respondents will complete follow-up surveys at 6-, 12- and 18-months post-infection.

**Discussion:**

These data can help identify which groups are most affected by PCC, and what health differences among demographic groups exist, as well as indicate potential barriers to care. These additional levels of granularity can inform public health action and help direct needed clinical care for patients.

**Supplementary Information:**

The online version contains supplementary material available at 10.1186/s12889-024-19772-4.

## Background

Post-COVID Conditions (PCC), or Long COVID, includes a wide range of symptoms and conditions that comprise new, returning, or ongoing health problems occurring at least 4 weeks after SARS-CoV-2 infection [1]. Over 200 symptoms are reported by individuals with PCC and can include fatigue and brain fog, shortness of breath, joint pain, and dizziness. PCC is associated with new conditions including neurological conditions, kidney damage or failure, diabetes, cardiovascular damage, and skin conditions [2, 3]. PCC can be debilitating and long lasting, impacting individuals’ ability to participate in daily activities and work [2, 3].

SARS-CoV-2 has caused over 104 million cases of COVID-19 in the U.S., and associated with over 6.3 million hospitalizations and 1.1 million deaths reported as of September 13, 2023. People continue to be infected by SARS-CoV-2, potentially leading to millions of people impacted by PCC since the start of the COVID-19 pandemic. Nationally, it is estimated that 1.3% of children and 6.9% of adults reported having Long COVID, or PCC, in 2022 [4]. Among adults with PCC, 1 in 4 report severe activity limitations [5]. While these estimates provide a snapshot of the number of people in the U.S. impacted, they only provide part of the picture.

To develop public health approaches and address health care needs of people with PCC, surveillance of PCC is necessary to provide estimates of the burden of PCC, track trends over time, and identify groups disproportionately impacted [6]. Public health surveillance is the systematic, ongoing collection, management, analysis, and interpretation of data, followed by dissemination of these data, to ultimately stimulate public health action [7]. One challenge in surveillance for PCC is that the estimates of burden often vary by the study approach and method [8]. These variable factors include whether the study population includes persons who had mild or asymptomatic infections or only persons who were hospitalized for COVID-19, whether the estimates are based on self-report of symptoms or from medical records, whether persons were followed at multiple time points from infection, whether the persons are representative of the general population, and many others. Surveillance that includes multiple approaches can begin to overcome these challenges [9].

The Tracking Post-COVID Conditions (Track PCC) project aims to address many of these factors by using both passive surveillance and active surveillance methods. Track PCC is a sentinel surveillance system to estimate PCC among all ages, across diverse populations at five geographic sites in the U.S. The purpose of this surveillance is to track and investigate the burden and impact of PCC within the U.S. to 1) estimate the incidence of PCC and track changes of incidence of PCC, 2) track and monitor the ongoing burden of PCC in individuals with history of previous SARS-CoV-2 infection including duration and severity of symptoms, and 3) describe social determinants of health and health disparities related to PCC. Passive surveillance methods will leverage electronic health records (EHR) and health information exchanges (HIE) to identify incidence of PCCs overall and for specific conditions. Active surveillance will use detailed surveys of persons infected with SARS-CoV-2 to estimate the prevalence of symptoms, symptom duration and severity, functional impairment, and impact on daily life. Passive and active surveillance will incorporate descriptions of risk factors for PCC, measures of social determinants of health and health disparities, and will include persons with both mild and severe COVID-19 illness. These data are essential for the development of public health strategies to reduce the burden of PCC in the U.S.

## Methods

### Overview

The Track PCC Surveillance Network is conducting passive surveillance with secondary analyses of data from multiple sources such as EHRs and HIEs (see below for details) and longitudinal active surveillance with primary data collection at 3-, 6-, 12- and 18-months post-SARS-CoV-2 infection. Passive surveillance data will retrospectively look to records from January 2020 and will continue to include new prospective data as it is generated. Active surveillance data will be collected prospectively beginning in November 2023.

This activity was initially reviewed by each site’s Institutional Review Board and CDC and deemed to be a public health surveillance activity and not research and was conducted consistent with applicable federal law and policy. (§ See e.g., 45 C.F.R. part 46.102(f), 46.102(k), 46.102(1)(2), 21 C.F.R. part 56; 42 U.S.C. §241 (d); 5 U.S.C. §552a; 44 U.S.C. §3501 et seq.) The Boise State University Institutional Review Board and the Temple University Institutional Review Board determined this project constitutes a PHSA. Subsequently, the University of Arizona Human Subjects Protection Program approved passive surveillance (#00001930) and active surveillance (#2003521636) as exempt research. The Indiana University Human Research Protection Program approved passive surveillance as exempt research (#16525) and active surveillance as expedited research (#20174).

### Settings and populations

**Fig. 1:**
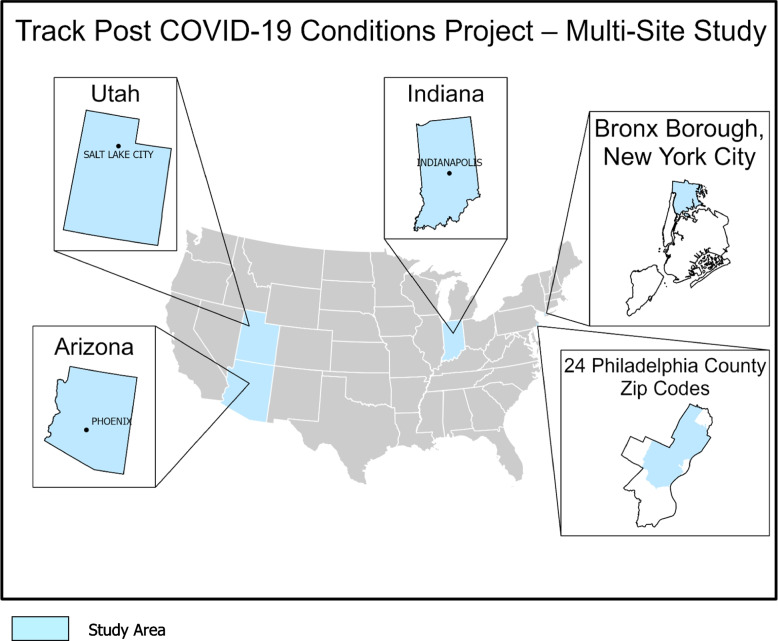
Track PCC Sites. Multi-Site Collaborators. Arizona: University of Arizona. Indiana: Indiana University. Bronx Borough, New York City: Boise State University, Bronx Regional Health Information Organization, Comagine Health. Utah: Boise State University, Comagine Health, Utah Health Information Network. Philadelphia County: Temple University

Track PCC is funded by the CDC through a Cooperative Agreement with Abt Global (data coordinating center) and four surveillance sites: Indiana University along with the Regenstrief Institute; Temple University; the University of Arizona; and the Comagine Health Collaborative, a collaborative between Boise State University, Comagine Health, Beyond HIE, Utah Health Information Network, and Bronx Regional Health Information Organization. Figure 1 shows a map of the United States with insets for each of the data collection sites. Site-specific population information is shown in Table 1.

**Table 1:** Population data for Track PCC sites

	**Arizona**	**Indiana**	**Philadelphia**^**a**^	**New York City **^**b**^	**Utah**
Total population	7,079,203	6,751,340	929,864	1,468,262	3,231,370
**Catchment Area**	Statewide	Statewide	NNE Philadelphia	Bronx borough	Statewide
**Population within Catchment Area**^**c**^					
% Male	49.9%	49.5%	47.8%	47.4%	50.6%
% Age 5 – 17 Years	17.0%	17.4%	15.6%	17.9%	21.7%
% Age 18 + Years	77.2%	76.4%	77.9%	75.1%	70.7%
% One race	89.6%	95.7%	95.0%	91.4%	94.4%
White	70.4%	81.2%	39.0%	19.9%	83.7%
Black or African American	4.5%	9.4%	39.0%	34.5%	1.2%
American Indian/Alaska Native	4.2%	0.2%	0.0%	0.8%	1.0%
Asian	3.3%	2.4%	7.0%	3.8%	2.3%
Some other race	7.1%	2.5%	10.0%	32.3%	6.1%
% Two or more races	10.4%	4.3%	5.0%	8.6%	5.6%
% Hispanic or Latino	31.9%	7.3%	18.0%	56.1%	14.4%
% Below Poverty	13.5%	12.5%	24.0%	26.5%	8.8%
**COVID-19 Data**^**d**^					
% > = 1 Vaccination	78.0%	65.0%	95.4%^e,f^	86.0%^e,g^	76.0%
COVID-19 Cases	2,486,671	2,033,879	234,984	516,827	1,099,978
COVID-19 Deaths	29,852	25,959	3,435	8,526	5,397
COVID-19 Case rate per 100,000^e^	35,126.40	30,125.60	25,270.80	35,199.90	34,040.60

*EHR* Electronic Health Record

^a^24 Select Zip Codes (i.e., 19111, 19114—19116, 19120—19126, 19129, 19130, 19132—19136, 19138, 19140, 19141, 19144, 19149, 19152)

^b^The Bronx Borough

^c^2022 American Community Survey 1-Year Estimates

^d^All Time Data pulled updated July 2023 https://usafacts.org/visualizations/coronavirus-covid-19-spread-map/

^e^Calculated

^f^https://opendataphilly.org/datasets/covid-19-vaccinations/ from October 2, 2023

^g^https://www.nyc.gov/site/doh/covid/covid-19-data-totals.page through September 11, 2023

### Passive surveillance methods

#### Data sources

Each site has access to patient-level data through health system electronic health records (EHR), Health Information Exchange (HIE) networks, and state or local health department COVID-19 vaccination and test result reporting systems. Data include ICD codes, laboratory results, vaccination information, COVID-19-associated clinical summaries and hospitalization records, and geographic indices.

#### Identification of COVID-19 cases and PCC

**Fig. 2:**
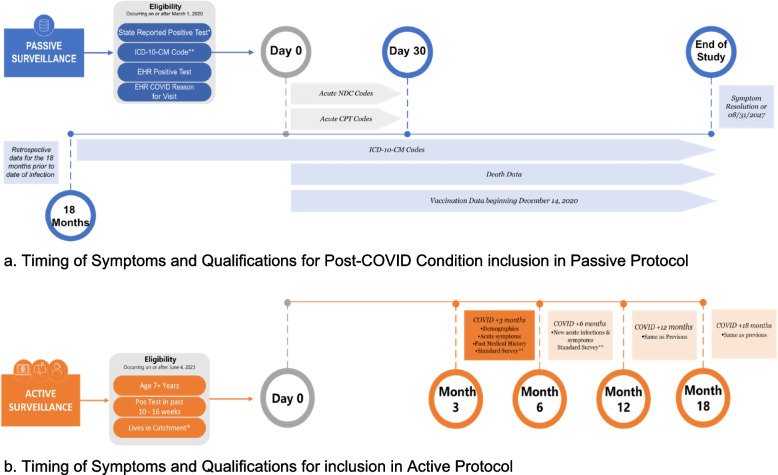
Timing of symptoms and qualifications of Post-COVID Conditions. **a** EHR: Electronic Health Record; CPT: Current Procedure Terminology Code; NDC: National Drug Code. *COVID-19 cases will be identified based on a positive laboratory test (i.e., nucleic acid- or antigen-based tests, using LOINC or SNOMED CT codes), encounter-level clinical diagnosis using ICD-10-CM codes, or report of a positive COVID-19 test in clinical records/notes. The date of an individual’s COVID-19 diagnosis is defined as Day 0. PCC is defined as the presence of one or more conditions 1–18 months after COVID-19 diagnosis that was not present prior to COVID-19 diagnosis. **See Additional file 1. **b** *List of Catchment areas: Arizona (statewide), Indiana (statewide), Philadelphia (i.e., 24 selected zip codes: 19111, 19114—19116, 19120—19126, 19129, 19130, 19132—19136, 19138, 19140, 19141, 19144, 19149, 19152), and the Bronx Borough of New York City. **Questionnaires associated with Resolution of symptoms, Treatments, Quality of life, Mental health, Activities of Daily Living

COVID-19 cases occurring between March 1, 2020, and June 30, 2027, will be identified from clinical records in each site’s catchment area. COVID-19 cases will be identified based on a positive laboratory test (i.e., nucleic acid- or antigen-based tests, using LOINC or SNOMED CT codes), encounter-level clinical diagnosis using ICD-10-CM codes, or report of a positive COVID-19 test in clinical records/notes. The date of an individual’s COVID-19 diagnosis is defined as Day 0. PCC is defined as the presence of one or more conditions 1–18 months after COVID-19 diagnosis that was not present prior to COVID-19 diagnosis (Fig. 2). Patients are required to have evidence of enrollment or contact with the medical system in the year prior to COVID-19 diagnosis to identify if a condition is newly diagnosed. PCC conditions will be identified via ICD-10 codes (Additional File 1). Surveillance is designed to be flexible and adaptable based on current scientific knowledge with definitions used to identify COVID-19 cases and PCC updated as needed and reviewed at least annually.

#### Data acquisition, collation, and harmonization

**Fig. 3:**
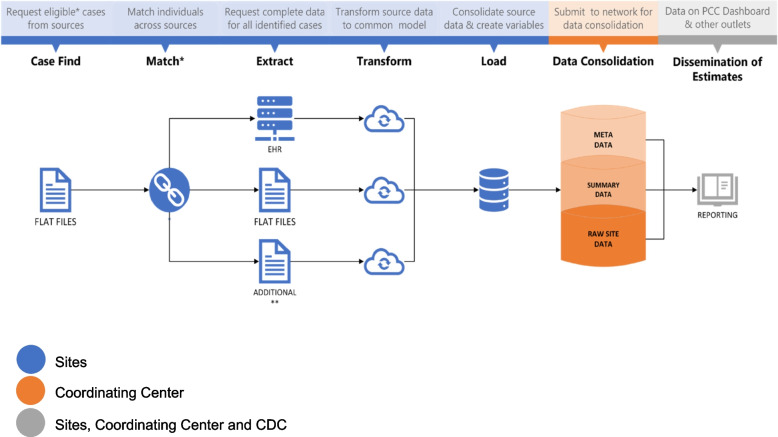
Data processing for Track PCC passive surveillance. PCC: Post-COVID Conditions; EHR: Electronic Health Record; COVID: CDC: Centers for Disease Control and Prevention. *Cases are matched across source data by name, date of birth and sex. Visits among cases that are duplicated across clinical and administrative sources are identified by date of visit.  **Vaccination, SARS-COV-2 testing, vital records, and census data

Data processing elements for passive surveillance are shown in Fig. 3. Following secure data acquisition, each site will consolidate their data and upload their files to a secure server where it can be accessed by the data coordinating center, Abt Global. Abt Global will perform data quality checks on all submitted files and then merge data from all sites to create combined datasets for analyses. Each site will also have access to their own site-specific datasets.

#### Estimates of PCC from passive surveillance

Main outcomes for the passive surveillance component will include estimates of incidence of PCC. Crude estimates will be calculated for each site at a range of follow-up periods following initial COVID-19 diagnosis. In addition, statistical methods will be used to generate prevalence estimates using demographics of the populations at each site. Incidence will be measured over time, enabling us to assess trends over time and SARS-CoV-2 variant periods. Estimates will be calculated for data from March 2020 through June 2027.

Additional analyses of interest include assessments of risk factors and disparities. Data on COVID-19 acute phase severity, pre-existing medical conditions, demographic, and community-level factors will be examined with respect to their association with PCC. We will further examine the association between individual-level and population-level social determinants of health and PCC burden. Where available, information on COVID-19 vaccination and acute COVID-19 treatments will be included to track the association between vaccination or treatment and PCC. Site-specific analyses may include examination of unique populations of interest, including Veterans, Native Americans, and Hispanics.

### Active surveillance methods

The active surveillance component comprises surveys of an estimated 4,000 SARS-CoV-2-positive individuals at 3-, 6-, 12-, and 18-months post-infection.

#### Active surveillance methods

Individuals ages 7 years and older living in each of the study sites’ catchment areas with evidence of positive SARS-CoV-2 test result (i.e., at-home test, or done by a clinician or laboratory) or a COVID-19 diagnosis will be eligible for enrollment. The target timing for the first survey is 3 months post-infection, with an acceptable range of 10–16 weeks post-infection to provide a window of time for recruitment, consent, and completion of the surveys. Follow-up surveys will be administered at 6-, 12-, and 18-months post-infection; each follow-up includes a 4- to 5-week window for survey completion. Participant eligibility is based on site-specific age targets, the ability to complete questionnaires in English or Spanish, being non-institutionalized, and meeting site-specific criteria for accessing the survey, such as an internet connection or telephone. Participation is voluntary and all participants must complete any site-specific consent and assent forms and allow for sharing of de-identified data. Following Institutional Review Board reviews, Track PCC’s active surveillance component received a public health surveillance activity determination from Boise State University and Temple University, whereas it was deemed exempt research by and the University of Arizona Human Subjects Protection Program and expedited research by the Indiana University Human Research Protection Program.

#### Recruitment targets

Beginning in Fall 2023, sites will recruit and enroll eligible participants at least quarterly over the course of approximately 2.5 years, with a goal of retaining a total of 1,000 individuals per site (*N* = 4,000) through the completion of the 18-month follow-up (See Table 2). Individuals who consent to participate in active surveillance will be enrolled in one of three age strata: 7–11 years, 12–17 years, or 18 years and older. Sites will aim to enroll consistently across quarters, with an annual goal of retaining ~ 444 participants per site. In consultation with the data coordinating center, sites established site-specific recruitment targets based on age, gender, and race/ethnicity.

**Table 2:** Active surveillance recruitment by site

	**Arizona**	**Indiana**	**Philadelphia**^**a**^	**New York City**^**b**^
**Recruitment strategies**				
Clinic in-person	x	x		x
Postal mail		x	x	x
Secure email		x	x	x
Flyers	x			x
Social media	x	x		x
Local events in-person	x			
Text messaging				
Phone calls			x	
**Survey administration**				
Self-administered	x	x	x	x
Assisted interview		x	x	x
Interviewer-administered			x	
**Eligible age groups**				
Children (7–11 years)		x	x	x
Youth (12–17 years)	x	x	x	x
Adults (18 + years)	x	x	x	x

*IRB* Institutional Review Board, *EHR* Electronic Health Record

^a^24 Select Zip Codes (i.e., 19111, 19114—19116, 19120—19126, 19129, 19130, 19132—19136, 19138, 19140, 19141, 19144, 19149, 19152)

^b^The Bronx Borough only; Utah is not participating in active surveillance

Participants will be considered enrolled after consenting or, where consent has been waived, completion of the baseline questionnaire 3 months post-infection. Participant retention is defined as completion of at least 3 of 4 surveys. Enrollment and retention will be monitored quarterly, and recruitment strategies will be adjusted accordingly to ensure adequate power for the planned statistical analyses.

#### Survey

Each adult and pediatric self-report survey (Additional files 2 and 3) includes previously validated instruments, as well as questions adapted from instruments widely used in studies of COVID-19, PCC, and other health topics [10–19]. The surveys include standardized multiple-choice and Likert-like scale items, as well as a limited number of open-ended questions. Adult and pediatric versions are similar except for age-specific validated instruments. Surveys were reviewed by all sites and the CDC for clarity, readability, plain language, and to ensure the reading level was no higher than eighth grade. The final English surveys were translated into Spanish. The 3-month baseline survey includes 105 items, with several items containing multiple questions. Table 3 provides a list of the topics that are being measured throughout the study, and Additional files 2 and 3 include the 3-month baseline questionnaire. Briefly, the surveys assess history of acute COVID-19 symptoms, ongoing symptoms and new diagnoses for multiple symptom groups (i.e., general; energy level, memory, and balance; digestive, ear, and eye; heart; well-being and mood; and other), symptom-specific assessments (i.e., fatigue, sleep disorders, physical function, cognitive function, impact on work/school activities), pre-existing medical conditions, COVID-19 vaccination information, and demographics. Modifications may be made to the 6-, 12-, and 18-month surveys based on data collected from the baseline survey, new topics of interest, or changes in our understanding of COVID-19 and PCC. The 3-month baseline questionnaire is expected to take 25–40 min to complete, and follow-up surveys are anticipated to take 20–30 min to complete.

**Table 3:** Active surveillance survey content

Survey sections	Instruments and citations
**History of acute COVID-19 symptoms**	Adapted from other instruments
**Ongoing and new symptoms—symptom groups**	
General	Adult—PROMIS Global Health v1.2^a^
	Pediatric – PROMIS Proxy Scale v1.0^b^
Energy level, memory, and balance	Adapted from other instruments
Digestive, ear, eye	Adapted from other instruments
Heart	Adapted from other instruments
Other	Adapted from other instruments
Well-being and mood	Adult/Pediatric—Warwick-Edinburgh Mental Well-being Scale (WEMWBS)^c,d^
**Symptom-specific assessments**	
Fatigue	Adult—PROMIS Fatigue 4a^e^
	Pediatric – PROMIS Fatigue 10a^f^
Physical function	Adult—PROMIS Physical Function Short Form 4a^g^
Cognitive function	PROMIS Cognitive Function 6a^h^
Impairment	Adult – World Health Organization Disability Assessment Schedule (WHODAS)^i^
Impact on work/school activites	PROMIS Satisfaction with Social Roles and Activities SF 4a^j^
**Demographics (at 3-month baseline)**	Adapted from other instruments
**New COVID-19 illnesses (at follow-up)**	Adapted from other instruments
**New Medical Conditions (at follow-up)**	Adapted from other instruments

General wellbeing assessment

^a^Adult – PROMIS Global Health v1.2 – Hays RD, Bjorner, J, Revicki RA, Spritzer KL, & Cella, D. Development of physical and mental health summary scores from the Patient Reported Outcomes Measurement Information System (PROMIS) global items. *Qual Life Res.* 2009;18(7):873–80

^b^Pediatric – PROMIS Proxy Scale v1.0 – Forrest C, Tucker C, Ravens-Sieberer U, Pratiwadi R, Moon J, Teneralli R, et al. Concurrent Validity of the PROMIS® Pediatric Global Health Measure. *Qual Life Res.* 2015;25(3):739–51

Mental wellbeing

^c^Adult – Marmara J, Zarate D, Vassallo J, Patten R, Stavropoulos V. Warwick Edinburgh Mental Well-Being Scale (WEMWBS): measurement invariance across genders and item response theory examination. *BMC Psychol*. 2022 Feb 18;10(1):31. https://doi.org/10.1186/s40359-022-00720-z. PMID: 35183262; PMCID: PMC8857792

^d^Pediatric – Clarke A, Friede T, Putz R. et al*.* Warwick-Edinburgh Mental Well-being Scale (WEMWBS): Validated for teenage school students in England and Scotland. A mixed methods assessment. *BMC Public Health.* 2011;11:487. https://doi.org/10.1186/1471-2458-11-487

Fatigue assessment

^e^Adult – PROMIS Fatigue 4a – Lai JS, Cella D, Choi SW, Junghaenel DU, Christodoulou C, Gershon R, & Stone A. How Item Banks and Their Application Can Influence Measurement Practice in Rehabilitation Medicine: A PROMIS Fatigue Item Bank Example. *Archives of Physical Medicine and Rehabilitation.* 2011;92(10 Supplement):S20-S27

^f^Pediatric – PROMIS Fatigue 10a – Sommer A, Grothus S, Grochowska K. et al*.* Assessing fatigue in children and adolescents: Psychometric validation of the German version of the PROMIS® Pediatric Short Form v2.0—Fatigue 10a in school children and pediatric chronic pain patients. *Qual Life Res.* 2022;31:1257–66. https://doi.org/10.1007/s11136-021-03032-8

Physical function assessment

^g^Adult – PROMIS Physical Function Short Form 4a—Jensen RE, Potosky AL, Reeve BB, Hahn E, Cella D, Fries J, Smith AW, Keegan TH, Wu XC, Paddock L, Moinpour CM. Validation of the PROMIS physical function measures in a diverse US population-based cohort of cancer patients. *Qual Life Res*. 2015 Oct;24(10):2333–44. https://doi.org/10.1007/s11136-015-0992-9. Epub 2015 May 3. PMID: 25935353; PMCID: PMC5079641

Cognitive function assessment

^h^Adult/Pediatric – PROMIS Cognitive Function 6a—Lai JS, Wagner LI, Jacobsen PB, & Cella D. Self-Reported Cognitive Concerns and Abilities: Two Sides of One Coin? *Psycho-Oncology*. 2014;23(10):1133–41.  10.1002/pon.3522

Impairment questionnaire

^i^Measuring Health and Disability: Manual for WHO Disability Assessment Schedule (WHODAS 2.0) / edited by TB Üstün, N Kostanjsek, S Chatterji, J Rehm. https://iris.who.int/bitstream/handle/10665/43974/9789241547598_eng.pdf?sequence=1

Impact on work/school activities

^j^Cella D, Choi SW, Condon DM, Schalet B, Hays RD, Rothrock NE, Yount S, Cook KF, Gershon RC, Amtmann D, DeWalt DA, Pilkonis PA, Stone AA, Weinfurt K, Reeve BB. PROMIS® Adult Health Profiles: Efficient Short-Form Measures of Seven Health Domains. *Value in Health*. 2019;22(5):537–44

#### Survey administration

To accommodate the varying needs of participants, multiple modes of survey administration will be used both across and within sites, including self-administered REDCap questionnaires, mailed questionnaires, and telephone-administered surveys. REDCap is a secure web platform for designing and managing online databases and surveys with functionality to export data in multiple common file formats.

Sites will send unique REDCap survey links and related information to participants via email or text message; periodic reminders will be sent to non-respondents at appropriate intervals. Mailed questionnaires may be sent to participants without an email address or cell phone or to individuals who do not respond to email or text messages. Mailed surveys will include a postage-paid business reply envelope. To boost retention, telephone interviewers may also contact participants by phone if other contact methods have not been successful within a specified timeframe.

A parent or legal guardian will be expected to complete surveys for all children less than 12 years; adolescents aged 12–17 years may elect to complete the questionnaires on their own or with parental assistance. Adults may request help to complete their surveys if needed (e.g., from a friend or family member). At the end of the survey, adolescent and adult participants will be asked to record whether they received assistance.

All sites will employ at least three reminder methods to increase survey completion; exact timing and frequency will vary by site. For sites using only REDCap, a combination of emails and text reminders will be sent on a regular schedule until the survey is completed or the maximum reminder attempts have been completed over the site-specific time frame. For sites using mail, telephone and/or in-person survey completion, reminders will be sent primarily by mail or telephone contact.

#### Incentives

All sites will provide financial incentives, including cash, e-gift cards, mailed gift cards, debit cards, or an opportunity to win additional incentives via lottery. Incentives will differ by site, taking into account institutional guidelines, target populations, and the need to ensure high response rates and retention.

#### Active surveillance estimates of PCC

Main outcomes for the active surveillance will include the prevalence of ongoing and new PCC symptoms overall and by symptom. In addition to crude estimates, survey sampling and statistical methods will be used to produce prevalence estimates that are representative of the SARS-CoV-2-positive population across the sites and over time by age group.

Additional analyses will include estimation of the association between COVID-19 vaccination, pre-existing medical conditions, individual-level demographics, and population-level social determinants on PCC over time.

## Dissemination of surveillance estimates

Timely data dissemination is a key component of sentinel surveillance for Track PCC. Incidence and prevalence of PCC, from both the passive and active surveillance, will be estimated at regular intervals to inform public health practitioners, researchers, and the general public about the burden of PCC in the U.S. Given many SARS-CoV-2 infections are not captured in EHRs or by health departments, PCC estimates will be reported both among persons with known COVID-19 diagnosis (i.e. COVID-19 cases as the denominator) and among the entire patient population or catchment area population (i.e. catchment area population as the denominator). Providing estimates with both denominators will provide a bounded range of incidence and prevalence of PCC in the population.

Dissemination may include publicly accessible websites, scientific peer-reviewed journal manuscripts, and presentations at regional, national, and international conferences.

## Discussion

Track PCC is uniquely poised to combine passive and active surveillance approaches to understand the incidence, prevalence, and burden of PCC among participants from five geographically diverse sites. The passive surveillance component will utilize the detailed clinical data available via EHR data to support estimates among a large sample of individuals, and the active surveillance component will facilitate a close-up look at self-reported symptoms, symptom persistence or resolution, and physical and mental well-being among a smaller sample. PCC include a broad range of conditions and symptoms that require a broad approach to surveillance to capture conditions and symptoms diagnosed in a healthcare setting, as well as those experienced by individuals that may not be captured in a healthcare setting.

### Strengths

This study has several strengths. The site partners represent geographically distinct and demographically diverse regions. The study will enroll children, adolescents, and adults, enabling estimates of incidence across a range of age groups. Additionally, the COVID-19 case definition includes self-report of approved at-home test, which overcomes limitations of other studies. Further, the five-year study design allows for enrollment over a long period of time, which facilitates the capture of data throughout multiple respiratory illness seasons and potentially across different SARS-CoV-2 variants. The design employs several recommended surveillance strategies [1], and the longitudinal nature of both the passive and active components permits us to assess symptom onset and the effects of PCC within the cohort over time. The use of EHR and HIE data from multiple sites for passive surveillance offers the opportunity to analyze PCC among a much larger cohort than would be possible via active surveillance alone, and the active surveillance component will enable us to analyze prevalence, resolution, and recurrence of individual symptoms over a span of 18 months. Active surveillance questionnaires will be administered via several modalities for participants’ convenience, and they are designed to gather comprehensive data not available via EHR or HIE without being overly burdensome for participants. The active surveillance system also allows for data collection amongst people who are not able to or choose not to seek medical care for their PCC symptoms, or whose symptoms are not captured in medical records.

### Limitations and possible solutions

This study also has several limitations. There is currently no standard case definition of PCC, and strategies for accurately identifying PCC in EHR data and based on self-report are still evolving [20]. However, this study is designed with this in mind to allow for flexibility with the definition of PCC and the ability to pivot if necessary to a new definition. Clinicians’ approaches to recognizing and coding PCC in the clinical setting are poorly understood, which could lead to misclassification within the passive surveillance data. The active surveillance component requires continued engagement from study participants and, therefore, ongoing retention efforts at each site. High attrition would hinder our ability to assess PCC over time; however, all sites have developed retention plans and aim to over-enroll participants to account for attrition. The active component may be affected by selection bias, as individuals with PCC or an interest in the topic may be more likely to participate. Modest incentives to all participants and reminders to participants who do not complete the questionnaires may encourage participation among a wide range of eligible participants. The ability to collect active surveillance data could be hampered by low incidence of SARS-CoV-2, making it more difficult to identify potentially eligible participants. Flexibility to increase recruitment efforts in the event of a surge of COVID-19 cases may be necessary to ensure adequate enrollment. In addition, this project follows the end of the public health emergency in May 2023. Thus, we anticipate a reduction in COVID-19 testing and tracking by state and local public health departments, which may impact sites’ ability to identify potential participants, as well as the overall denominator of COVID-19 cases. Further, this may equate to fewer people with milder infections being included in the data. Finally, the patient population may not be representative of the entire U.S. and the results may not be generalizable to the U.S. population. However, the diverse nature of participating sites should reduce that concern and analyses will be able to account for individual- and area-level characteristics.

In conclusion, Track PCC, with its robust passive and active surveillance components, will increase our understanding of PCC incidence, risk factors, and impact in large and diverse cohorts using both EHR and self-report data.

### Supplementary Information

Supplementary Material 1.

Supplementary Material 2.

Supplementary Material 3.

## Data Availability

This manuscript describes the design and active and passive surveillance protocols for this multi-site study – no data are currently available for analysis. Subsequent publications that analyze data and provide study findings will provide information about the availability of data. The ICD codes for passive surveillance and baseline active surveillance questionnaires for adults and children are included in the article and its additional files.
