# Supplementary material for: Tracking the burden, distribution, and impact of Post-COVID conditions in diverse populations for children, adolescents, and adults (Track PCC): passive and active surveillance protocols
Source: BMC Public Health. 2024 Aug 29;24:2345. doi: 10.1186/s12889-024-19772-4 (PMC11360551; doi:10.1186/s12889-024-19772-4)
Supplement: Supplementary file 1 — Supplementary Material 1. [file 12889_2024_19772_MOESM1_ESM.pdf]

Appendix A. ICD-10-CM codes used in initial PCC case definitions

**Condition category**	**ICD codes (includes subordinate categories unless otherwise noted)**
Acute myocardial infarction	I21-I22
Acute pulmonary embolism	I26
Cardiac dysrhythmias	I47-I49, R000-R009
Cerebrovascular disease	G46, H341, I60-I68
Circulatory signs and symptoms	R030, R0989
Coronary atherosclerosis and other heart disease	I20, I24, I251, I256-I259, Z9861, R570, R010-R012
Rheumatic heart disease	I05-I09
Congestive heart failure	I502-I503
Hypertension	I10, I11, I15, I16
Myocarditis and cardiomyopathy	A3681, B3320, B3322, B334, B5881, I255, I40-I43, J1082, J1182
Dermatologic conditions	B001, L20, L50
Type I diabetes	E10
Type 2 diabetes	E11
Digestive system disorders	K58-K59, K929, R194, R198
Esophageal disorders	K20-K23
Coagulation and hemorrhagic disorders	D473, D65, D68-D69, D7582-D7583, M362
Phlebitis; thrombophlebitis and thromboembolism	I824-I827
Muscle disorders	G719, G728-G729, M60-M63
Musculoskeletal pain, not low back pain	M255-M256, M546, M5489, M549, M791, M796, M797
Myoneural Disorders	G70
Foreign Body Granulomas	M602, M79
Anxiety disorders	F064, F40-F41, F431, F930
Ataxia / Trouble Walking	G26, R26-R27, R282
Autonomic Dysfunction	G90, I951, R55
Cognitive disorders	F01-F03, F05, G30, G31, G310, G934, R400, R41, R44,
Encephalitis	A85, A86, G04,G05, R29
Headache	G43-G44, R51
Mood disorders	F0630, F3170, F3172, F3174, F3178, F32-F34, F39, F631, F632, F634, R4584
Peripheral Nerve Disorders	G50-G59, G61-G65, M5481
Seizures	G40-G41
Sleep Disorders	F51, G47, R063
Substance use disorders	F10-F19
Symptoms and signs involving cognition, perception, emotional state and behavior	R401-R402, R410, R4182-R41844, R42, R44-R46
Kidney disease	I12-I13 ,N17-N19, R880
Liver disease	B18, I85, I864, K70-K76
Asthma	J452-J459
Respiratory disease, not asthma	J84, J960-J961, R060-R0682
Respiratory symptoms	R04-R07, R09
Hearing loss and disturbances	H90A, H912, H919, H9323-H9329
Smell and taste disturbances	R43, R438-R439
Visual disturbances	H5310, H5314, H538
Malaise and fatigue	G933, R53, R531, R538
Nutritional deficiency	E40-E46, E640, R634

Multisystem Inflammatory Syndrome	M3581
Autoimmune diseases	D510, D6861, D75822, D869, E0500, E063, G35, K5090-K51919, K900,L129,L40, L63, L80,L931,L95, M064,M069, M081, M3130-M3131, M316, M32-M353, M45, M468, M479
PCC-defining condition	U099
