# Supplementary material for: Tracking the burden, distribution, and impact of Post-COVID conditions in diverse populations for children, adolescents, and adults (Track PCC): passive and active surveillance protocols
Source: BMC Public Health. 2024 Aug 29;24:2345. doi: 10.1186/s12889-024-19772-4 (PMC11360551; doi:10.1186/s12889-024-19772-4)
Supplement: Supplementary file 2 — Supplementary Material 2. [file 12889_2024_19772_MOESM2_ESM.pdf]

On behalf of the Centers for Disease Control and Prevention (CDC), thank you for participating in this ongoing survey. Your responses will be important in helping us understand how COVID-19 affects the public.

Your information will be kept confidential. Please answer each question to the best of your ability.

Your participation is voluntary. You may skip any question and may stop participating at any time. This survey should take around 25-40 minutes to finish.

Please fill in the circle that matches your answer with a black pen. Answer each question to the best of your ability. If you are unsure about how to answer a question, please give the best answer you can.

Marking Instructions: **Correct:** ● **Incorrect:** ⊗ ✓ ◒

COVID-19 Testing

1. Thinking about your most recent COVID-19 illness, what was the date you tested positive for COVID-19? You may have had a positive test using an at-home test kit, a test at a clinic, urgent care or your doctor's office, or a lab test like a PCR. If you cannot remember the exact date, provide your best guess.

   | | | / | | | / | 2 | 0 | | |

   Month Day Year

   (Mark here if uncertain of day: ○)

2. Have you ever tested positive for COVID-19 before this most recent COVID-19 illness?

   ○ Yes

   ○ No → **Skip to #4**

   ○ Not sure → **Skip to #4**

3. If yes, about how long ago did your previous COVID-19 illness(es) occur? **Mark all that apply**.

   ○ 0-3 months ago
   ○ 4-6 months ago
   ○ 7-12 months ago
   ○ More than 12 months ago
   ○ Unsure

Acute COVID-19

For this section, think about your most recent positive COVID-19 test or diagnosis. This is the date you entered for question #1. Do your best to remember your experience.

4. Did you have any of the following new symptoms around the time of your COVID-19 diagnosis? Please only choose the symptoms that you remember having during the week **before** or the first four weeks **after** your positive test or diagnosis. **Mark all that apply**:
   - ○ Fever
   - ○ Chills
   - ○ Changes in sense of taste
   - ○ Changes in sense of smell
   - ○ Fatigue, tiredness, or weakness
   - ○ Cough
   - ○ Shortness of breath or difficulty breathing (feelings of tightness in the chest, not having enough air, or being "hungry" for air)
   - ○ Chest pain
   - ○ Runny nose or congestion
   - ○ Muscle or body aches
   - ○ Headache
   - ○ Sore throat
   - ○ Nausea or vomiting
   - ○ Stomach or abdominal pain
   - ○ Diarrhea
   - ○ Other, please specify: ______________________________
   - ○ No symptoms → **Skip to #6**

5. How much difficulty did these new symptoms cause with your day-to-day work, school, or other regular activities?
   - ○ No difficulty
   - ○ Mild difficulty
   - ○ Moderate difficulty
   - ○ Severe difficulty
   - ○ Extreme difficulty, or unable to do these activities

6. Did you receive any medical treatment or see a doctor, nurse, or other health professional for your most recent COVID-19 illness? This is the date you entered for question #1.
   - ○ Yes
   - ○ No → **Skip to #8**

7. What type(s) of medical care did you receive? **Mark all that apply**.
   - ○ I was treated in an outpatient setting (for instance, urgent care, clinic, doctor's office, or health department)
   - ○ I had one or more telehealth visits but did not see a health provider in person
   - ○ I visited the emergency department but was not admitted to the hospital
   - ○ I was admitted to the hospital but not the intensive care unit
   - ○ I was admitted to the intensive care unit
   - ○ Other, please specify: ______________________________

8. Did you take any medications to treat your most recent COVID-19 illness? **Mark all that apply**.
   - ○ Paxlovid (nirmatrelvir/ritonavir)
   - ○ Veklury (remdesivir)
   - ○ Monoclonal antibodies such as Lagevrio (monulpiravir)
   - ○ Over the counter pain or fever relief, such Tylenol or Advil
   - ○ Other, please specify: ______________________________
   - ○ I do not know which medication(s) I took
   - ○ I did not take any medications

Post COVID-19 Conditions

The following questions ask about different types of symptoms you may have had since your most recent COVID-19 illness. This is the date you entered for question #1.

Think about your most recent COVID-19 illness. In the following questions, choose the symptoms you had that lasted **4 weeks or more.** These can be symptoms that have continued since your illness, symptoms that got better over time and then returned, or new symptoms. Do not choose symptoms that you had before your COVID-19 illness.

If you have not experienced any of the symptoms in a list, please mark the circle at the end of the list. There is space after the last list to add any other symptoms you experienced that are not listed.

9. **General Symptoms**

	Did you have this symptom for 4 weeks or longer?	When did this symptom start?	Are you still experiencing this symptom?	If you are no longer experiencing this symptom, when did it get better?
Persistent fever	○ Yes<br>○ No	○ When I got COVID-19<br>○ Less than 1 month after<br>○ 1-2 months after<br>○ More than 2 months after<br>○ Not sure	○ Yes<br>○ No	○ Within the past few days<br>○ 1-2 weeks ago<br>○ 3-4 weeks ago<br>○ Between 1 and 2 months ago<br>○ More than 2 months ago<br>○ Not sure
Persistent chills	○ Yes<br>○ No	○ When I got COVID-19<br>○ Less than 1 month after<br>○ 1-2 months after<br>○ More than 2 months after<br>○ Not sure	○ Yes<br>○ No	○ Within the past few days<br>○ 1-2 weeks ago<br>○ 3-4 weeks ago<br>○ Between 1 and 2 months ago<br>○ More than 2 months ago<br>○ Not sure

	Did you have this symptom for 4 weeks or longer?	When did this symptom start?	Are you still experiencing this symptom?	If you are no longer experiencing this symptom, when did it get better?
Changes in sense of taste	○ Yes<br>○ No	○ When I got COVID-19<br>○ Less than 1 month after<br>○ 1-2 months after<br>○ More than 2 months after<br>○ Not sure	○ Yes<br>○ No	○ Within the past few days<br>○ 1-2 weeks ago<br>○ 3-4 weeks ago<br>○ Between 1 and 2 months ago<br>○ More than 2 months ago<br>○ Not sure
Changes in sense of smell	○ Yes<br>○ No	○ When I got COVID-19<br>○ Less than 1 month after<br>○ 1-2 months after<br>○ More than 2 months after<br>○ Not sure	○ Yes<br>○ No	○ Within the past few days<br>○ 1-2 weeks ago<br>○ 3-4 weeks ago<br>○ Between 1 and 2 months ago<br>○ More than 2 months ago<br>○ Not sure
Fatigue, tiredness, or weakness	○ Yes<br>○ No	○ When I got COVID-19<br>○ Less than 1 month after<br>○ 1-2 months after<br>○ More than 2 months after<br>○ Not sure	○ Yes<br>○ No	○ Within the past few days<br>○ 1-2 weeks ago<br>○ 3-4 weeks ago<br>○ Between 1 and 2 months ago<br>○ More than 2 months ago<br>○ Not sure
Cough	○ Yes<br>○ No	○ When I got COVID-19<br>○ Less than 1 month after<br>○ 1-2 months after<br>○ More than 2 months after<br>○ Not sure	○ Yes<br>○ No	○ Within the past few days<br>○ 1-2 weeks ago<br>○ 3-4 weeks ago<br>○ Between 1 and 2 months ago<br>○ More than 2 months ago<br>○ Not sure

	Did you have this symptom for 4 weeks or longer?	When did this symptom start?	Are you still experiencing this symptom?	If you are no longer experiencing this symptom, when did it get better?
Shortness of breath or difficulty breathing (feelings of tightness in the chest, not having enough air, or being "hungry" for air)	○ Yes<br>○ No	○ When I got COVID-19<br>○ Less than 1 month after<br>○ 1-2 months after<br>○ More than 2 months after<br>○ Not sure	○ Yes<br>○ No	○ Within the past few days<br>○ 1-2 weeks ago<br>○ 3-4 weeks ago<br>○ Between 1 and 2 months ago<br>○ More than 2 months ago<br>○ Not sure
Chest pain	○ Yes<br>○ No	○ When I got COVID-19<br>○ Less than 1 month after<br>○ 1-2 months after<br>○ More than 2 months after<br>○ Not sure	○ Yes<br>○ No	○ Within the past few days<br>○ 1-2 weeks ago<br>○ 3-4 weeks ago<br>○ Between 1 and 2 months ago<br>○ More than 2 months ago<br>○ Not sure
Runny nose or congestion	○ Yes<br>○ No	○ When I got COVID-19<br>○ Less than 1 month after<br>○ 1-2 months after<br>○ More than 2 months after<br>○ Not sure	○ Yes<br>○ No	○ Within the past few days<br>○ 1-2 weeks ago<br>○ 3-4 weeks ago<br>○ Between 1 and 2 months ago<br>○ More than 2 months ago<br>○ Not sure
Muscle or body aches	○ Yes<br>○ No	○ When I got COVID-19<br>○ Less than 1 month after<br>○ 1-2 months after<br>○ More than 2 months after<br>○ Not sure	○ Yes<br>○ No	○ Within the past few days<br>○ 1-2 weeks ago<br>○ 3-4 weeks ago<br>○ Between 1 and 2 months ago<br>○ More than 2 months ago<br>○ Not sure

	Did you have this symptom for 4 weeks or longer?	When did this symptom start?	Are you still experiencing this symptom?	If you are no longer experiencing this symptom, when did it get better?
Headache	○ Yes<br>○ No	○ When I got COVID-19<br>○ Less than 1 month after<br>○ 1-2 months after<br>○ More than 2 months after<br>○ Not sure	○ Yes<br>○ No	○ Within the past few days<br>○ 1-2 weeks ago<br>○ 3-4 weeks ago<br>○ Between 1 and 2 months ago<br>○ More than 2 months ago<br>○ Not sure
Sore throat	○ Yes<br>○ No	○ When I got COVID-19<br>○ Less than 1 month after<br>○ 1-2 months after<br>○ More than 2 months after<br>○ Not sure	○ Yes<br>○ No	○ Within the past few days<br>○ 1-2 weeks ago<br>○ 3-4 weeks ago<br>○ Between 1 and 2 months ago<br>○ More than 2 months ago<br>○ Not sure
Nausea or vomiting	○ Yes<br>○ No	○ When I got COVID-19<br>○ Less than 1 month after<br>○ 1-2 months after<br>○ More than 2 months after<br>○ Not sure	○ Yes<br>○ No	○ Within the past few days<br>○ 1-2 weeks ago<br>○ 3-4 weeks ago<br>○ Between 1 and 2 months ago<br>○ More than 2 months ago<br>○ Not sure
Stomach or abdominal pain	○ Yes<br>○ No	○ When I got COVID-19<br>○ Less than 1 month after<br>○ 1-2 months after<br>○ More than 2 months after<br>○ Not sure	○ Yes<br>○ No	○ Within the past few days<br>○ 1-2 weeks ago<br>○ 3-4 weeks ago<br>○ Between 1 and 2 months ago<br>○ More than 2 months ago<br>○ Not sure

	Did you have this symptom for 4 weeks or longer?	When did this symptom start?	Are you still experiencing this symptom?	If you are no longer experiencing this symptom, when did it get better?
Diarrhea	○ Yes<br>○ No	○ When I got COVID-19<br>○ Less than 1 month after<br>○ 1-2 months after<br>○ More than 2 months after<br>○ Not sure	○ Yes<br>○ No	○ Within the past few days<br>○ 1-2 weeks ago<br>○ 3-4 weeks ago<br>○ Between 1 and 2 months ago<br>○ More than 2 months ago<br>○ Not sure
I did not experience any of these symptoms.	○ I did not experience any of these symptoms.			

10. Again, think about your most recent COVID-19 illness (the date you entered for question #1). Choose the symptoms you had that lasted **4 weeks or more**. These can be symptoms that continued since your illness, symptoms that got better over time and then returned, or new symptoms. Do not choose symptoms that you had before your COVID-19 illness.

Energy Level, Memory, and Balance Symptoms

	Did you have this symptom for 4 weeks or longer?	When did this symptom start?	Are you still experiencing this symptom?	If you are no longer experiencing this symptom, when did it get better?
Symptoms that get worse after even mild physical activity or mental effort, also known as post-exertional malaise	○ Yes<br>○ No	○ When I got COVID-19<br>○ Less than 1 month after<br>○ 1-2 months after<br>○ More than 2 months after<br>○ Not sure	○ Yes<br>○ No	○ Within the past few days<br>○ 1-2 weeks ago<br>○ 3-4 weeks ago<br>○ Between 1 and 2 months ago<br>○ More than 2 months ago<br>○ Not sure
Problems sleeping	○ Yes<br>○ No	○ When I got COVID-19<br>○ Less than 1 month after<br>○ 1-2 months after<br>○ More than 2 months after<br>○ Not sure	○ Yes<br>○ No	○ Within the past few days<br>○ 1-2 weeks ago<br>○ 3-4 weeks ago<br>○ Between 1 and 2 months ago<br>○ More than 2 months ago<br>○ Not sure

	Did you have this symptom for 4 weeks or longer?	When did this symptom start?	Are you still experiencing this symptom?	If you are no longer experiencing this symptom, when did it get better?
Problems speaking or communicating	○ Yes<br>○ No	○ When I got COVID-19<br>○ Less than 1 month after<br>○ 1-2 months after<br>○ More than 2 months after<br>○ Not sure	○ Yes<br>○ No	○ Within the past few days<br>○ 1-2 weeks ago<br>○ 3-4 weeks ago<br>○ Between 1 and 2 months ago<br>○ More than 2 months ago<br>○ Not sure
Difficulty thinking clearly or concentrating, forgetfulness, memory loss, or 'brain fog'	○ Yes<br>○ No	○ When I got COVID-19<br>○ Less than 1 month after<br>○ 1-2 months after<br>○ More than 2 months after<br>○ Not sure	○ Yes<br>○ No	○ Within the past few days<br>○ 1-2 weeks ago<br>○ 3-4 weeks ago<br>○ Between 1 and 2 months ago<br>○ More than 2 months ago<br>○ Not sure
Problems with balance or movement	○ Yes<br>○ No	○ When I got COVID-19<br>○ Less than 1 month after<br>○ 1-2 months after<br>○ More than 2 months after<br>○ Not sure	○ Yes<br>○ No	○ Within the past few days<br>○ 1-2 weeks ago<br>○ 3-4 weeks ago<br>○ Between 1 and 2 months ago<br>○ More than 2 months ago<br>○ Not sure
Dizziness, lightheadedness, or fainting	○ Yes<br>○ No	○ When I got COVID-19<br>○ Less than 1 month after<br>○ 1-2 months after<br>○ More than 2 months after<br>○ Not sure	○ Yes<br>○ No	○ Within the past few days<br>○ 1-2 weeks ago<br>○ 3-4 weeks ago<br>○ Between 1 and 2 months ago<br>○ More than 2 months ago<br>○ Not sure
I did not experience any of these symptoms.	○ I did not experience any of these symptoms.			

11. Again, thinking about the time between your most recent COVID-19 illness and today, please select any ongoing, recurring, or new symptoms that have lasted **4 weeks or more**.

Digestive, Ear, and Eye Symptoms

	Did you have this symptom for 4 weeks or longer?	When did this symptom start?	Are you still experiencing this symptom?	If you are no longer experiencing this symptom, when did it get better?
Appetite changes (for example: eating more than normal, eating less than normal)	○ Yes<br>○ No	○ When I got COVID-19<br>○ Less than 1 month after<br>○ 1-2 months after<br>○ More than 2 months after<br>○ Not sure	○ Yes<br>○ No	○ Within the past few days<br>○ 1-2 weeks ago<br>○ 3-4 weeks ago<br>○ Between 1 and 2 months ago<br>○ More than 2 months ago<br>○ Not sure
Problems swallowing or chewing	○ Yes<br>○ No	○ When I got COVID-19<br>○ Less than 1 month after<br>○ 1-2 months after<br>○ More than 2 months after<br>○ Not sure	○ Yes<br>○ No	○ Within the past few days<br>○ 1-2 weeks ago<br>○ 3-4 weeks ago<br>○ Between 1 and 2 months ago<br>○ More than 2 months ago<br>○ Not sure
Reflux or heartburn	○ Yes<br>○ No	○ When I got COVID-19<br>○ Less than 1 month after<br>○ 1-2 months after<br>○ More than 2 months after<br>○ Not sure	○ Yes<br>○ No	○ Within the past few days<br>○ 1-2 weeks ago<br>○ 3-4 weeks ago<br>○ Between 1 and 2 months ago<br>○ More than 2 months ago<br>○ Not sure
Constipation	○ Yes<br>○ No	○ When I got COVID-19<br>○ Less than 1 month after<br>○ 1-2 months after<br>○ More than 2 months after<br>○ Not sure	○ Yes<br>○ No	○ Within the past few days<br>○ 1-2 weeks ago<br>○ 3-4 weeks ago<br>○ Between 1 and 2 months ago<br>○ More than 2 months ago<br>○ Not sure

	Did you have this symptom for 4 weeks or longer?	When did this symptom start?	Are you still experiencing this symptom?	If you are no longer experiencing this symptom, when did it get better?
Tingling or numbness in any part of body	○ Yes<br>○ No	○ When I got COVID-19<br>○ Less than 1 month after<br>○ 1-2 months after<br>○ More than 2 months after<br>○ Not sure	○ Yes<br>○ No	○ Within the past few days<br>○ 1-2 weeks ago<br>○ 3-4 weeks ago<br>○ Between 1 and 2 months ago<br>○ More than 2 months ago<br>○ Not sure
Ringing in ears (tinnitus)	○ Yes<br>○ No	○ When I got COVID-19<br>○ Less than 1 month after<br>○ 1-2 months after<br>○ More than 2 months after<br>○ Not sure	○ Yes<br>○ No	○ Within the past few days<br>○ 1-2 weeks ago<br>○ 3-4 weeks ago<br>○ Between 1 and 2 months ago<br>○ More than 2 months ago<br>○ Not sure
Eye symptoms (pink eye, conjunctivitis, red eyes, excessive tearing, etc.)	○ Yes<br>○ No	○ When I got COVID-19<br>○ Less than 1 month after<br>○ 1-2 months after<br>○ More than 2 months after<br>○ Not sure	○ Yes<br>○ No	○ Within the past few days<br>○ 1-2 weeks ago<br>○ 3-4 weeks ago<br>○ Between 1 and 2 months ago<br>○ More than 2 months ago<br>○ Not sure
I did not experience any of these symptoms.	○ I did not experience any of these symptoms.			

12. Again, thinking about the time between your most recent COVID-19 illness and today, please select any ongoing, recurring, or new symptoms that have lasted **4 weeks or more**.

Heart Symptoms

	Did you have this symptom for 4 weeks or longer?	When did this symptom start?	Are you still experiencing this symptom?	If you are no longer experiencing this symptom, when did it get better?
Palpitations (heart racing or pounding)	○ Yes<br>○ No	○ When I got COVID-19<br>○ Less than 1 month after<br>○ 1-2 months after<br>○ More than 2 months after<br>○ Not sure	○ Yes<br>○ No	○ Within the past few days<br>○ 1-2 weeks ago<br>○ 3-4 weeks ago<br>○ Between 1 and 2 months ago<br>○ More than 2 months ago<br>○ Not sure
Arrythmia (irregular heartbeat)	○ Yes<br>○ No	○ When I got COVID-19<br>○ Less than 1 month after<br>○ 1-2 months after<br>○ More than 2 months after<br>○ Not sure	○ Yes<br>○ No	○ Within the past few days<br>○ 1-2 weeks ago<br>○ 3-4 weeks ago<br>○ Between 1 and 2 months ago<br>○ More than 2 months ago<br>○ Not sure
Faster than normal heart rate	○ Yes<br>○ No	○ When I got COVID-19<br>○ Less than 1 month after<br>○ 1-2 months after<br>○ More than 2 months after<br>○ Not sure	○ Yes<br>○ No	○ Within the past few days<br>○ 1-2 weeks ago<br>○ 3-4 weeks ago<br>○ Between 1 and 2 months ago<br>○ More than 2 months ago<br>○ Not sure
Slower than normal heart rate	○ Yes<br>○ No	○ When I got COVID-19<br>○ Less than 1 month after<br>○ 1-2 months after<br>○ More than 2 months after<br>○ Not sure	○ Yes<br>○ No	○ Within the past few days<br>○ 1-2 weeks ago<br>○ 3-4 weeks ago<br>○ Between 1 and 2 months ago<br>○ More than 2 months ago<br>○ Not sure

	Did you have this symptom for 4 weeks or longer?	When did this symptom start?	Are you still experiencing this symptom?	If you are no longer experiencing this symptom, when did it get better?
New high blood pressure	○ Yes<br>○ No	○ When I got COVID-19<br>○ Less than 1 month after<br>○ 1-2 months after<br>○ More than 2 months after<br>○ Not sure	○ Yes<br>○ No	○ Within the past few days<br>○ 1-2 weeks ago<br>○ 3-4 weeks ago<br>○ Between 1 and 2 months ago<br>○ More than 2 months ago<br>○ Not sure
Chest pain or pressure	○ Yes<br>○ No	○ When I got COVID-19<br>○ Less than 1 month after<br>○ 1-2 months after<br>○ More than 2 months after<br>○ Not sure	○ Yes<br>○ No	○ Within the past few days<br>○ 1-2 weeks ago<br>○ 3-4 weeks ago<br>○ Between 1 and 2 months ago<br>○ More than 2 months ago<br>○ Not sure
I did not experience any of these symptoms.	○ I did not experience any of these symptoms.			

13. Again, thinking about the time between your most recent COVID-19 illness and today, please select any ongoing, recurring, or new symptoms that have lasted **4 weeks or more**.

Other Symptoms

	Did you have this symptom for 4 weeks or longer?	When did this symptom start?	Are you still experiencing this symptom?	If you are no longer experiencing this symptom, when did it get better?
Bruising/ bleeding easily	○ Yes<br>○ No	○ When I got COVID-19<br>○ Less than 1 month after<br>○ 1-2 months after<br>○ More than 2 months after<br>○ Not sure	○ Yes<br>○ No	○ Within the past few days<br>○ 1-2 weeks ago<br>○ 3-4 weeks ago<br>○ Between 1 and 2 months ago<br>○ More than 2 months ago<br>○ Not sure

	Did you have this symptom for 4 weeks or longer?	When did this symptom start?	Are you still experiencing this symptom?	If you are no longer experiencing this symptom, when did it get better?
Changes in menstrual cycle (for example: irregular periods, shorter or longer cycles)	○ Yes → **Skip to 'Hair Loss' two rows below.**<br>○ No<br>○ Not applicable	○ When I got COVID-19<br>○ Less than 1 month after<br>○ 1-2 months after<br>○ More than 2 months after<br>○ Not sure	○ Yes<br>○ No	○ Within the past few days<br>○ 1-2 weeks ago<br>○ 3-4 weeks ago<br>○ Between 1 and 2 months ago<br>○ More than 2 months ago<br>○ Not sure
Erectile dysfunction	○ Yes<br>○ No<br>○ Not applicable	○ When I got COVID-19<br>○ Less than 1 month after<br>○ 1-2 months after<br>○ More than 2 months after<br>○ Not sure	○ Yes<br>○ No	○ Within the past few days<br>○ 1-2 weeks ago<br>○ 3-4 weeks ago<br>○ Between 1 and 2 months ago<br>○ More than 2 months ago<br>○ Not sure
Hair loss	○ Yes<br>○ No	○ When I got COVID-19<br>○ Less than 1 month after<br>○ 1-2 months after<br>○ More than 2 months after<br>○ Not sure	○ Yes<br>○ No	○ Within the past few days<br>○ 1-2 weeks ago<br>○ 3-4 weeks ago<br>○ Between 1 and 2 months ago<br>○ More than 2 months ago<br>○ Not sure
Joint swelling	○ Yes<br>○ No	○ When I got COVID-19<br>○ Less than 1 month after<br>○ 1-2 months after<br>○ More than 2 months after<br>○ Not sure	○ Yes<br>○ No	○ Within the past few days<br>○ 1-2 weeks ago<br>○ 3-4 weeks ago<br>○ Between 1 and 2 months ago<br>○ More than 2 months ago<br>○ Not sure

	Did you have this symptom for 4 weeks or longer?	When did this symptom start?	Are you still experiencing this symptom?	If you are no longer experiencing this symptom, when did it get better?
Joint pain	○ Yes<br>○ No	○ When I got COVID-19<br>○ Less than 1 month after<br>○ 1-2 months after<br>○ More than 2 months after<br>○ Not sure	○ Yes<br>○ No	○ Within the past few days<br>○ 1-2 weeks ago<br>○ 3-4 weeks ago<br>○ Between 1 and 2 months ago<br>○ More than 2 months ago<br>○ Not sure
Skin changes or rash (hive-like rashes, discoloration, etc.)	○ Yes<br>○ No	○ When I got COVID-19<br>○ Less than 1 month after<br>○ 1-2 months after<br>○ More than 2 months after<br>○ Not sure	○ Yes<br>○ No	○ Within the past few days<br>○ 1-2 weeks ago<br>○ 3-4 weeks ago<br>○ Between 1 and 2 months ago<br>○ More than 2 months ago<br>○ Not sure
Unintentional weight loss	○ Yes<br>○ No	○ When I got COVID-19<br>○ Less than 1 month after<br>○ 1-2 months after<br>○ More than 2 months after<br>○ Not sure	○ Yes<br>○ No	○ Within the past few days<br>○ 1-2 weeks ago<br>○ 3-4 weeks ago<br>○ Between 1 and 2 months ago<br>○ More than 2 months ago<br>○ Not sure
I did not experience any of these symptoms.	○ I did not experience any of these symptoms.			

14. Again, thinking about the time between your most recent COVID-19 illness and today, please select any ongoing, recurring, or new symptoms that have lasted **4 weeks or more**.

Well-Being and Mood Symptoms

	Did you have this symptom for 4 weeks or longer?	When did this symptom start?	Are you still experiencing this symptom?	If you are no longer experiencing this symptom, when did it get better?
Feeling anxious, restless, or on edge; unable to stop worrying; trouble relaxing; irritability	○ Yes<br>○ No	○ When I got COVID-19<br>○ Less than 1 month after<br>○ 1-2 months after<br>○ More than 2 months after<br>○ Not sure	○ Yes<br>○ No	○ Within the past few days<br>○ 1-2 weeks ago<br>○ 3-4 weeks ago<br>○ Between 1 and 2 months ago<br>○ More than 2 months ago<br>○ Not sure
Feelings of sadness, hopelessness, or loss of interest or pleasure in activities	○ Yes<br>○ No	○ When I got COVID-19<br>○ Less than 1 month after<br>○ 1-2 months after<br>○ More than 2 months after<br>○ Not sure	○ Yes<br>○ No	○ Within the past few days<br>○ 1-2 weeks ago<br>○ 3-4 weeks ago<br>○ Between 1 and 2 months ago<br>○ More than 2 months ago<br>○ Not sure
Having nightmares, unwanted memories of trauma	○ Yes<br>○ No	○ When I got COVID-19<br>○ Less than 1 month after<br>○ 1-2 months after<br>○ More than 2 months after<br>○ Not sure	○ Yes<br>○ No	○ Within the past few days<br>○ 1-2 weeks ago<br>○ 3-4 weeks ago<br>○ Between 1 and 2 months ago<br>○ More than 2 months ago<br>○ Not sure
Mood swings	○ Yes<br>○ No	○ When I got COVID-19<br>○ Less than 1 month after<br>○ 1-2 months after<br>○ More than 2 months after<br>○ Not sure	○ Yes<br>○ No	○ Within the past few days<br>○ 1-2 weeks ago<br>○ 3-4 weeks ago<br>○ Between 1 and 2 months ago<br>○ More than 2 months ago<br>○ Not sure
I did not experience any of these symptoms.	○ I did not experience any of these symptoms.			

15. Thinking about the time between your most recent COVID-19 illness and today, **did you have any other symptoms that lasted 4 weeks or more**? These can include ongoing symptoms from the time of COVID-19 illness, symptoms that got better and then returned, or new symptoms. Do not include symptoms that you usually had before your COVID-19 illness.

	Did you have this symptom for 4 weeks or longer?	When did this symptom start?	Are you still experiencing this symptom?	If you are no longer experiencing this symptom, when did it get better?
I did not experience any other symptoms.	○ I did not experience any other symptoms.			
Other, please specify: ____________ ____________ ____________ ____________	○ Yes<br>○ No	○ When I got COVID-19<br>○ Less than 1 month after<br>○ 1-2 months after<br>○ More than 2 months after<br>○ Not sure	○ Yes<br>○ No	○ Within the past few days<br>○ 1-2 weeks ago<br>○ 3-4 weeks ago<br>○ Between 1 and 2 months ago<br>○ More than 2 months ago<br>○ Not sure
Other, please specify: ____________ ____________ ____________ ____________	○ Yes<br>○ No	○ When I got COVID-19<br>○ Less than 1 month after<br>○ 1-2 months after<br>○ More than 2 months after<br>○ Not sure	○ Yes<br>○ No	○ Within the past few days<br>○ 1-2 weeks ago<br>○ 3-4 weeks ago<br>○ Between 1 and 2 months ago<br>○ More than 2 months ago<br>○ Not sure
Other, please specify: ____________ ____________ ____________ ____________	○ Yes<br>○ No	○ When I got COVID-19<br>○ Less than 1 month after<br>○ 1-2 months after<br>○ More than 2 months after<br>○ Not sure	○ Yes<br>○ No	○ Within the past few days<br>○ 1-2 weeks ago<br>○ 3-4 weeks ago<br>○ Between 1 and 2 months ago<br>○ More than 2 months ago<br>○ Not sure

16. Are there any other symptoms you haven’t already mentioned that have gotten WORSE since your most recent COVID-19 illness?
   - ○ Yes
   - ○ No → **Skip to #18**

17. Please describe these symptoms.

If no symptoms were marked or mentioned in questions #9 - #17, → skip to #21.

18. Did you see a doctor, nurse, or other health professional or visit a hospital or other health facility (such as an urgent care, clinic, doctor's office, or health department) for any of these symptom(s) you marked in questions #9-#17?
   - ○ Yes
   - ○ No
   - ○ Prefer not to answer

19. How much difficulty did/do these symptoms cause with your day-to-day work, school, or other regular activities?
   - ○ No difficulty
   - ○ Mild difficulty
   - ○ Moderate
   - ○ Severe difficulty
   - ○ Extreme difficulty, or unable to do these activities

20. Have you been told by a health care provider you have Long COVID, Post-COVID Conditions, or Post-Acute Sequelae of COVID-19 (PASC)?
   - ○ Yes
   - ○ No
   - ○ Not sure
   - ○ Prefer not to answer

Past Medical History

21. Do you **currently** smoke **cigarettes:**
   - ○ Every day
   - ○ Some days
   - ○ Not at all
   - ○ Don’t know
   - ○ Prefer not to answer

22. Have you smoked at least **100 cigarettes** in your entire life?
   - ○ Yes
   - ○ No
   - ○ Don't know
   - ○ Prefer not to answer

23. Do you currently smoke **cigars, or a pipe, or do you use e-cigarettes or any vaping product** on a daily basis?
   - ○ Yes → **Skip to #25**
   - ○ No
   - ○ Prefer not to answer

24. In the past, did you smoke **cigars, or a pipe, or did you use e-cigarettes or any vaping product** on a daily basis?
   - ○ Yes
   - ○ No
   - ○ Prefer not to answer

25. Below are some common health conditions. Have you ever been told by a doctor or health care professional that you have or had any of these conditions? If so, did these conditions first occur before or after your most recent COVID-19 illness? **Mark the circles that apply for each condition.**

	Have you ever been told that you had this condition? ***Mark all that apply***	Did this condition first occur before or after your first positive COVID-19 test result/diagnosis?	Do you still have this condition?
Chronic lung disease, such as asthma or COPD (chronic obstructive pulmonary disease)	○ Yes<br>○ No	○ Before<br>○ After	○ Yes<br>○ No
A mental health disorder, such as anxiety or depression	○ Yes<br>○ No	○ Before<br>○ After	○ Yes<br>○ No
An immune system disorder, such as Parkinson's, systemic lupus, multiple sclerosis, chronic fatigue syndrome, rheumatoid arthritis, HIV, having been a recipient of an organ or bone marrow transplant, being on immunosuppressive medication, missing your spleen, or severe combined immunoglobulin deficiency (SCID)	○ Yes<br>○ No	○ Before<br>○ After	

	Have you ever been told that you had this condition? ***Mark all that apply***	Did this condition first occur before or after your first positive COVID-19 test result/diagnosis?	Do you still have this condition?
Cancer, *including* solid organ cancer and leukemia/lymphoma; *excluding* non-melanoma skin cancer	○ Yes<br>○ No	○ Before<br>○ After	○ Yes<br>○ No
Chronic kidney disease, such as chronic renal failure, end-stage renal failure or renal failure requiring dialysis	○ Yes<br>○ No	○ Before<br>○ After	
Chronic liver disease, such as fatty liver, steatohepatitis, alcoholic liver disease or chronic hepatitis C	○ Yes<br>○ No	○ Before<br>○ After	
Chronic headaches or migraines	○ Yes<br>○ No	○ Before<br>○ After	○ Yes<br>○ No
Heart disease, other cardiovascular disease, or heart attack	○ Yes<br>○ No	○ Before<br>○ After	
High blood pressure	○ Yes<br>○ No	○ Before<br>○ After	○ Yes<br>○ No
High cholesterol	○ Yes<br>○ No	○ Before<br>○ After	○ Yes<br>○ No
Diabetes, **Check type:**<br>☐ Type 1 ☐ Type 2<br>☐ Not sure	○ Yes<br>○ No	○ Before<br>○ After	○ Yes<br>○ No
Gestational diabetes	○ Yes<br>○ No<br>○ Not applicable	○ Before<br>○ After	○ Yes<br>○ No
Chronic pain	○ Yes<br>○ No	○ Before<br>○ After	○ Yes<br>○ No
Stroke	○ Yes<br>○ No	○ Before<br>○ After	
Obesity	○ Yes<br>○ No	○ Before<br>○ After	○ Yes<br>○ No
Other, please specify:<br>____________________	○ Yes<br>○ No	○ Before<br>○ After	○ Yes<br>○ No
Other, please specify:<br>____________________	○ Yes<br>○ No	○ Before<br>○ After	○ Yes<br>○ No
None of these	○ None of these		

26. Are you currently pregnant?
- ○ Yes
- ○ No
- ○ Not applicable

27. Do you have any other health conditions or concerns that have been made worse by COVID-19?
- ○ Yes, please specify: ____
- ○ No

28. What is your height?

		Feet			Inches

- ○ Don't know
- ○ Prefer not to answer

29. What is your weight?

			Pounds

- ○ Don't know
- ○ Prefer not to answer

COVID-19 Vaccination

The following questions are about COVID-19 vaccinations you may have received. You may find it helpful to check your vaccination records when answering these questions. If you don't have your records available, please use your best guess.

30. Have you ever received a COVID-19 vaccine?
- ○ Yes
- ○ No → **Skip to #36**
- ○ Don't know → **Skip to #36**
- ○ Prefer not to answer → **Skip to #36**

31. Did you receive a COVID-19 vaccine **before** your COVID-19 illness you entered for question #1?
- ○ Yes
- ○ No → **Skip to #33**
- ○ Don't know → **Skip to #33**
- ○ Prefer not to answer → **Skip to #33**

32. When was your most recent COVID-19 vaccine *before* your COVID-19 illness you entered for question #1?

				2	0		
Month		Day		Year			

- ○ Don't know
- ○ Prefer not to answer

33. Did you receive a COVID-19 vaccination **after** you had COVID-19 you entered for question #1?
- ○ Yes
- ○ No → **Skip to #35**
- ○ Don't know → **Skip to #35**
- ○ Prefer not to answer → **Skip to #35**

34. When was your most recent COVID-19 vaccination?

				2	0		
Month		Day		Year			

- ○ Don't know
- ○ Prefer not to answer

35. How many total COVID-19 shots have you had?

☐☐ Total COVID-19 shots

- ○ Don't know
- ○ Prefer not to answer

General Wellbeing Assessment

36. In general, would you say your health is:
- ○ Excellent
- ○ Very good
- ○ Good
- ○ Fair
- ○ Poor

37. In general, would you say your quality of life is:
- ○ Excellent
- ○ Very good
- ○ Good
- ○ Fair
- ○ Poor

38. In general, how would you rate your physical health?
- ○ Excellent
- ○ Very good
- ○ Good
- ○ Fair
- ○ Poor

39. In general, how would you rate your mental health, including your mood and your ability to think?
- ○ Excellent
- ○ Very good
- ○ Good
- ○ Fair
- ○ Poor

40. In general, how would you rate your satisfaction with your social activities and relationships?
- ○ Excellent
- ○ Very good
- ○ Good
- ○ Fair
- ○ Poor

41. In general, please rate how well you carry out your usual social activities and roles. This includes activities at home, at work and in your community, and responsibilities as a parent, child, spouse, employee, friend, etc.
- ○ Excellent
- ○ Very good
- ○ Good
- ○ Fair
- ○ Poor

42. To what extent are you able to carry out your everyday physical activities such as walking, climbing stairs, carrying groceries, or moving a chair?
- ○ Completely
- ○ Mostly
- ○ Moderately
- ○ A little
- ○ Not at all

43. In the past 7 days, how often have you been bothered by emotional problems such as feeling anxious, depressed, or irritable?
- ○ Never
- ○ Rarely
- ○ Sometimes
- ○ Often
- ○ Always

44. In the past 7 days, how would you rate your fatigue on average?
- ○ None
- ○ Mild
- ○ Moderate
- ○ Severe
- ○ Very severe

45. In the past 7 days, how would you rate your pain on average?

No pain										Worst pain imaginable
0	1	2	3	4	5	6	7	8	9	10
○	○	○	○	○	○	○	○	○	○	○

Mental Wellbeing

Below are some statements about feelings and thoughts. **Please mark the answer that best describes your experience of each statement over the last 14 days**.

	None of the time	Rarely	Some of the time	Often	All of the time
46. I've been feeling optimistic about the future	○	○	○	○	○
47. I've been feeling useful	○	○	○	○	○
48. I've been feeling relaxed	○	○	○	○	○
49. I've been feeling interested in other people	○	○	○	○	○
50. I've had energy to spare	○	○	○	○	○
51. I've been dealing with problems well	○	○	○	○	○
52. I've been thinking clearly	○	○	○	○	○
53. I've been feeling good about myself	○	○	○	○	○
54. I've been feeling close to other people	○	○	○	○	○
55. I've been feeling confident	○	○	○	○	○
56. I've been able to make up my own mind about things	○	○	○	○	○
57. I've been feeling loved	○	○	○	○	○
58. I've been interested in new things	○	○	○	○	○
59. I've been feeling cheerful	○	○	○	○	○

Impairment Questionnaire

These questions ask about **difficulties you may be experiencing because of health conditions**. Health conditions include diseases or illnesses, other health problems that may be short or long lasting, injuries, mental or emotional problems, and problems with alcohol or drugs.

Think back over the **past 30 days** and answer these questions and think about how much difficulty you had doing the following activities.

In the past 30 days, how much difficulty did you have in:

	None	Mild	Moderate	Severe	Extreme or cannot do
60. Standing for long periods such as 30 minutes?	○	○	○	○	○
61. Taking care of your household responsibilities?	○	○	○	○	○
62. Learning a new task, for example, learning how to get to a new place?	○	○	○	○	○
63. How much of a problem did you have joining in community activities (for example, festivities, religious, or other activities) in the same way as anyone else can?	○	○	○	○	○
64. How much have you been emotionally affected by your health problems?	○	○	○	○	○
65. Concentrating on doing something for ten minutes?	○	○	○	○	○
66. Walking a long distance such as a mile?	○	○	○	○	○
67. Washing your whole body?	○	○	○	○	○
68. Getting dressed?	○	○	○	○	○
69. Dealing with people you do not know?	○	○	○	○	○
70. Maintaining a friendship?	○	○	○	○	○
71. Your day-to-day work?	○	○	○	○	○

72. Overall, in the past 30 days, how many days were one or more of these difficulties present?

☐☐ Days

73. In the past 30 days, how many days were you totally unable to carry out your usual activities or work because of any health condition?

☐☐ Days

74. In the past 30 days, not counting the days that you were totally unable, for how many days did you cut back or reduce your usual activities or work because of any health condition?

☐☐ Days

Fatigue Assessment

Please respond to each question or statement by **marking one answer for each item**.

During the past 7 days:

	Not at all	A little bit	Somewhat	Quite a bit	Very much
75. I feel fatigued	○	○	○	○	○
76. I have trouble starting things because I am tired	○	○	○	○	○
77. How run-down did you feel on average?	○	○	○	○	○
78. How fatigued were you on average?	○	○	○	○	○

Cognitive Function Assessment

In the past 7 days:

	Never	Rarely (Once)	Sometimes (Two or three times)	Often (About once a day)	Very often (Several times a day)
79. My thinking has been slow...	○	○	○	○	○
80. It has seemed like my brain was not working as well as usual...	○	○	○	○	○
81. I have had to work harder than usual to keep track of what I was doing...	○	○	○	○	○
82. I have had trouble shifting back and forth between different activities that require thinking...	○	○	○	○	○
83. I have had trouble concentrating...	○	○	○	○	○
84. I have had to work really hard to pay attention or I would make a mistake...	○	○	○	○	○

Impact on Work/School Assessment

85. In the past 7 days: Did you take time off work or school due to illness?

- ○ Yes
- ○ No → **Skip to #87**
- ○ Not applicable – I don't go to work or school → **Skip to #87**
- ○ Prefer not to answer → **Skip to #87**

86. How many days did you take off in the past 7 days?

☐ Days

87. In the past 7 days, did someone else take time off work or school to care for you due to your illness?
- ○ Yes
- ○ No → **Skip to #89**
- ○ Prefer not to answer → **Skip to #89**

88. In the past 7 days, how many days did someone else take time off to care for you?

☐ Days

Physical Function Assessment

Thinking about the time since your most recent COVID-19 diagnosis, please respond to each question **by marking one answer for each item.**

	Without any difficulty	With a little difficulty	With some difficulty	With much difficulty	Unable to do
89. Are you able to do chores such as vacuuming or yard work?	○	○	○	○	○
90. Are you able to go up and down stairs at a normal pace?	○	○	○	○	○
91. Are you able to go for a walk of at least 15 minutes?	○	○	○	○	○
92. Are you able to run errands and shop?	○	○	○	○	○

Demographics

93. What sex were you assigned at birth on your original birth certificate?
- ○ Male
- ○ Female
- ○ Prefer not to answer

94. Do you currently describe yourself as:
- ○ Male
- ○ Female
- ○ Transgender female
- ○ Transgender male
- ○ I use a different term, please specify:________________________________
- ○ Prefer not to answer

95. What is your date of birth?

☐☐ Month ☐☐ Day ☐☐☐☐ Year

96. What is your ethnicity?
- ○ Hispanic
- ○ Non-Hispanic
- ○ Prefer not to answer

97. What is your race? **Select all that apply.**
- ○ Asian or Asian American
- ○ Black or African American
- ○ Native Hawaiian or other Pacific Islander
- ○ American Indian or Alaska Native
- ○ White or Caucasian
- ○ Other race, please specify:____
- ○ Prefer not to answer

98. How many people currently live in your household, including you?

[ ][ ]	Total number people living in household

- ○ I live in a group setting such as a nursing home. → **Skip to #100**
- ○ Prefer not to answer → **Skip to #100**

99. How many children under 18 years of age currently live in your household?

[ ][ ]	Number of children under 18 living in household

100. What is the highest degree or level of school that you have completed?
- ○ Never attended school or only attended kindergarten
- ○ Grades 1- 8
- ○ Grades 9-11
- ○ High school graduate or GED
- ○ College, 1-3 years, or some college, associate's degree, or technical school
- ○ College, 4 years or more, or college graduate
- ○ Post-graduate education or professional degree (e.g., MA, PhD, MD, DDS)
- ○ Prefer not to answer

101. Select the employment category that best describes you. Are you currently:
- ○ Employed for wages or salary
- ○ Self-employed or business owner
- ○ Out of work for 1 year or more
- ○ Out of work for less than 1 year
- ○ A homemaker
- ○ A student
- ○ Retired
- ○ Unable to work
- ○ Other, please specify: ____
- ○ Prefer not to answer

102. What kind(s) of health insurance or health care coverage do you have? **Mark all that apply.**
- ○ Medicare (Including Medicare Advantage plans)
- ○ Medicaid (Names of these plans may differ by state, for example Medi Cal in California)
- ○ Private (either employer-provided or an individually purchased plan, including plans purchased under the Affordable Care Act and COBRA, and temporary health insurance)
- ○ Military insurance, such as Tricare
- ○ Veterans Administration
- ○ Other, please specify: ____
- ○ No health care coverage of any type
- ○ Not sure
- ○ Prefer not to answer

103. What is your annual household income from all sources?
- ○ $0 to $24,999
- ○ $25,000 to $49,999
- ○ $50,000 to $74,999
- ○ $75,000 to $99,999
- ○ $100,000 to $149,999
- ○ $150,000 or more
- ○ Don't know/Not sure
- ○ Prefer not to answer

104. Did you receive assistance from someone (for example, a family member or caregiver) to complete this survey?
- ○ Yes
- ○ No → **Skip to #105**

104a. Who assisted you in completing this survey?

105. Has any member of your household taken a survey as part of this project?
- ○ Yes
- ○ No
- ○ I’m not sure

Thank you for completing this questionnaire.

Please return the questionnaire in the postage-paid envelope provided.

We will mail you your $50 ‘thank you’ once we receive your completed questionnaire.

You will hear from us in about three months to complete a follow-up questionnaire.

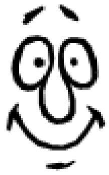

Temple University
