# Supplementary material for: Tracking the burden, distribution, and impact of Post-COVID conditions in diverse populations for children, adolescents, and adults (Track PCC): passive and active surveillance protocols
Source: BMC Public Health. 2024 Aug 29;24:2345. doi: 10.1186/s12889-024-19772-4 (PMC11360551; doi:10.1186/s12889-024-19772-4)
Supplement: Supplementary file 3 — Supplementary Material 3. [file 12889_2024_19772_MOESM3_ESM.pdf]

NOTE: The questionnaire items are identical for those ages 7-17 years; however, the orientation is slightly different as 12-17 years are encouraged to complete themselves with help from their parent/guardian so questions asked about "you/your" whereas for ages 7-11 years, the parent/guardian completes the questionnaire with input from their child.

This survey is intended for children ages 7-11. Children 7-11 years old should have their parent/guardian answer the questions below on their behalf. We encourage the parent/guardian and child to work together to complete the survey. Some questions might be easier for the child to answer and some might be easier for the adult to answer.

Your child's information will be kept confidential. Please answer each question to the best of your knowledge.

Your participation is voluntary. You may skip any question and may stop participating at any time. This survey should take around 25-40 minutes to finish.

Please fill in the circle that matches your answer with a black pen. Answer each question to the best of your ability. If you are unsure about how to answer a question, please give the best answer you can.

Marking Instructions: **Correct:** ● **Incorrect:** ⊗ ✓ ◒

COVID-19 Testing

1. Thinking about your child's most recent COVID-19 illness, what was the date they tested positive for COVID-19? They may have had a positive test using an at-home test kit, a test at a clinic, urgent care or their doctor's office, or a lab test like a PCR. If you cannot remember the exact date, provide your best guess.

Month		Day		Year			
				2	0		

(Mark here if uncertain of day: ○)

2. Has your child ever tested positive for COVID-19 before this most recent COVID-19 illness?
   - ○ Yes
   - ○ No → **Skip to #4**
   - ○ Not sure → **Skip to #4**

3. If yes, about how long ago did your child's previous COVID-19 illness(es) occur? **Mark all that apply**.
   - ○ 0-3 months ago
   - ○ 4-6 months ago
   - ○ 7-12 months ago
   - ○ More than 12 months ago
   - ○ Unsure

Acute COVID-19

For this section, think about your child's most recent positive COVID-19 test or diagnosis. This is the date you entered for question #1. Do your best to remember your child's experience.

4. Did your child have any of the following new symptoms around the time of their COVID-19 diagnosis? Please only choose the symptoms that you remember your child having during the week **before** or the first four weeks **after** their positive test or diagnosis. **Mark all that apply**:
   - ○ Fever
   - ○ Chills
   - ○ Changes in sense of taste
   - ○ Changes in sense of smell
   - ○ Fatigue, tiredness, or weakness
   - ○ Cough
   - ○ Shortness of breath or difficulty breathing (feelings of tightness in the chest, not having enough air, or being "hungry" for air)
   - ○ Chest pain
   - ○ Runny nose or congestion
   - ○ Muscle or body aches
   - ○ Headache
   - ○ Sore throat
   - ○ Nausea or vomiting
   - ○ Stomach or abdominal pain
   - ○ Diarrhea
   - ○ Other, please specify: ____
   - ○ No symptoms → **Skip to #6**

5. How much difficulty did these new symptoms cause with your child's daily life, school, or other regular activities?
   - ○ No difficulty
   - ○ Mild difficulty
   - ○ Moderate difficulty
   - ○ Severe difficulty
   - ○ Extreme difficulty, or my child couldn't do these activities

6. Did your child get any medical treatment for their most recent COVID-19 illness? This is the date you entered for question #1. Medical care can be from your child's doctor/pediatrician, nurse, or another healthcare professional.
   - ○ Yes
   - ○ No → **Skip to #8**

7. What type(s) of medical care did your child receive? **Mark all that apply**.
   - ○ My child was treated in an outpatient setting (for instance, urgent care, clinic, doctor's office, or health department)
   - ○ My child had one or more telehealth visits but did not see a health provider in person
   - ○ My child visited the emergency department but was not admitted to the hospital
   - ○ My child was admitted to the hospital but not the intensive care unit
   - ○ My child was admitted to the intensive care unit
   - ○ Other, please specify: ____

8. Did your child take any medications to treat their most recent COVID-19 illness? **Mark all that apply**.
   - ○ Paxlovid (nirmatrelvir/ritonavir)
   - ○ Veklury (remdesivir)
   - ○ Over the counter pain or fever relief, such Tylenol or Advil
   - ○ Other, please specify: ____
   - ○ I do not know which medication(s) my child took
   - ○ My child did not take any medications.

Post COVID-19 Conditions

The following questions ask about different types of symptoms your child may have had since their most recent COVID-19 illness. This is the date you entered for question #1.

Think about your child's most recent COVID-19 illness. In the table below, choose the symptoms your child had that lasted **4 weeks or more.** These can be symptoms that have continued since your child's illness, symptoms that got better over time and then returned, or new symptoms. Do not choose symptoms that your child had before their COVID-19 illness.

If your child has not experienced any of the symptoms in a table, please mark the circle at the bottom of the table. There is space after the last table to add any other symptoms your child experienced that are not listed.

9. **General Symptoms**

	Did child have symptom for 4 weeks or longer?	When did this symptom start?	Are they still experiencing this symptom?	If they are no longer experiencing this symptom, when did it get better?
Persistent fever	○ Yes<br>○ No	○ When they got COVID-19<br>○ Less than 1 month after<br>○ 1-2 months after<br>○ More than 2 months after<br>○ Not sure	○ Yes<br>○ No	○ Within the past few days<br>○ 1-2 weeks ago<br>○ 3-4 weeks ago<br>○ Between 1 and 2 months ago<br>○ More than 2 months ago<br>○ Not sure
Persistent chills	○ Yes<br>○ No	○ When they got COVID-19<br>○ Less than 1 month after<br>○ 1-2 months after<br>○ More than 2 months after<br>○ Not sure	○ Yes<br>○ No	○ Within the past few days<br>○ 1-2 weeks ago<br>○ 3-4 weeks ago<br>○ Between 1 and 2 months ago<br>○ More than 2 months ago<br>○ Not sure

	Did child have symptom for 4 weeks or longer?	When did this symptom start?	Are they still experiencing this symptom?	If they are no longer experiencing this symptom, when did it get better?
Changes in sense of taste	○ Yes<br>○ No	○ When they got COVID-19<br>○ Less than 1 month after<br>○ 1-2 months after<br>○ More than 2 months after<br>○ Not sure	○ Yes<br>○ No	○ Within the past few days<br>○ 1-2 weeks ago<br>○ 3-4 weeks ago<br>○ Between 1 and 2 months ago<br>○ More than 2 months ago<br>○ Not sure
Changes in sense of smell	○ Yes<br>○ No	○ When they got COVID-19<br>○ Less than 1 month after<br>○ 1-2 months after<br>○ More than 2 months after<br>○ Not sure	○ Yes<br>○ No	○ Within the past few days<br>○ 1-2 weeks ago<br>○ 3-4 weeks ago<br>○ Between 1 and 2 months ago<br>○ More than 2 months ago<br>○ Not sure
Fatigue, tiredness, or weakness	○ Yes<br>○ No	○ When they got COVID-19<br>○ Less than 1 month after<br>○ 1-2 months after<br>○ More than 2 months after<br>○ Not sure	○ Yes<br>○ No	○ Within the past few days<br>○ 1-2 weeks ago<br>○ 3-4 weeks ago<br>○ Between 1 and 2 months ago<br>○ More than 2 months ago<br>○ Not sure
Cough	○ Yes<br>○ No	○ When they got COVID-19<br>○ Less than 1 month after<br>○ 1-2 months after<br>○ More than 2 months after<br>○ Not sure	○ Yes<br>○ No	○ Within the past few days<br>○ 1-2 weeks ago<br>○ 3-4 weeks ago<br>○ Between 1 and 2 months ago<br>○ More than 2 months ago<br>○ Not sure

	Did child have symptom for 4 weeks or longer?	When did this symptom start?	Are they still experiencing this symptom?	If they are no longer experiencing this symptom, when did it get better?
Shortness of breath or difficulty breathing (feelings of tightness in the chest, not having enough air, or being "hungry" for air)	○ Yes<br>○ No	○ When they got COVID-19<br>○ Less than 1 month after<br>○ 1-2 months after<br>○ More than 2 months after<br>○ Not sure	○ Yes<br>○ No	○ Within the past few days<br>○ 1-2 weeks ago<br>○ 3-4 weeks ago<br>○ Between 1 and 2 months ago<br>○ More than 2 months ago<br>○ Not sure
Chest pain	○ Yes<br>○ No	○ When they got COVID-19<br>○ Less than 1 month after<br>○ 1-2 months after<br>○ More than 2 months after<br>○ Not sure	○ Yes<br>○ No	○ Within the past few days<br>○ 1-2 weeks ago<br>○ 3-4 weeks ago<br>○ Between 1 and 2 months ago<br>○ More than 2 months ago<br>○ Not sure
Runny nose or congestion	○ Yes<br>○ No	○ When they got COVID-19<br>○ Less than 1 month after<br>○ 1-2 months after<br>○ More than 2 months after<br>○ Not sure	○ Yes<br>○ No	○ Within the past few days<br>○ 1-2 weeks ago<br>○ 3-4 weeks ago<br>○ Between 1 and 2 months ago<br>○ More than 2 months ago<br>○ Not sure
Muscle or body aches	○ Yes<br>○ No	○ When they got COVID-19<br>○ Less than 1 month after<br>○ 1-2 months after<br>○ More than 2 months after<br>○ Not sure	○ Yes<br>○ No	○ Within the past few days<br>○ 1-2 weeks ago<br>○ 3-4 weeks ago<br>○ Between 1 and 2 months ago<br>○ More than 2 months ago<br>○ Not sure

	Did child have symptom for 4 weeks or longer?	When did this symptom start?	Are they still experiencing this symptom?	If they are no longer experiencing this symptom, when did it get better?
Headache	○ Yes<br>○ No	○ When they got COVID-19<br>○ Less than 1 month after<br>○ 1-2 months after<br>○ More than 2 months after<br>○ Not sure	○ Yes<br>○ No	○ Within the past few days<br>○ 1-2 weeks ago<br>○ 3-4 weeks ago<br>○ Between 1 and 2 months ago<br>○ More than 2 months ago<br>○ Not sure
Sore throat	○ Yes<br>○ No	○ When they got COVID-19<br>○ Less than 1 month after<br>○ 1-2 months after<br>○ More than 2 months after<br>○ Not sure	○ Yes<br>○ No	○ Within the past few days<br>○ 1-2 weeks ago<br>○ 3-4 weeks ago<br>○ Between 1 and 2 months ago<br>○ More than 2 months ago<br>○ Not sure
Nausea or vomiting	○ Yes<br>○ No	○ When they got COVID-19<br>○ Less than 1 month after<br>○ 1-2 months after<br>○ More than 2 months after<br>○ Not sure	○ Yes<br>○ No	○ Within the past few days<br>○ 1-2 weeks ago<br>○ 3-4 weeks ago<br>○ Between 1 and 2 months ago<br>○ More than 2 months ago<br>○ Not sure
Stomach or abdominal pain	○ Yes<br>○ No	○ When they got COVID-19<br>○ Less than 1 month after<br>○ 1-2 months after<br>○ More than 2 months after<br>○ Not sure	○ Yes<br>○ No	○ Within the past few days<br>○ 1-2 weeks ago<br>○ 3-4 weeks ago<br>○ Between 1 and 2 months ago<br>○ More than 2 months ago<br>○ Not sure

	Did child have symptom for 4 weeks or longer?	When did this symptom start?	Are they still experiencing this symptom?	If they are no longer experiencing this symptom, when did it get better?
Diarrhea	○ Yes<br>○ No	○ When they got COVID-19<br>○ Less than 1 month after<br>○ 1-2 months after<br>○ More than 2 months after<br>○ Not sure	○ Yes<br>○ No	○ Within the past few days<br>○ 1-2 weeks ago<br>○ 3-4 weeks ago<br>○ Between 1 and 2 months ago<br>○ More than 2 months ago<br>○ Not sure
My child did not experience any of these symptoms.	○ My child did not experience any of these symptoms.			

10. Again, think about your child's most recent COVID-19 illness (the date you entered for question #1). In the table below, choose the symptoms your child had that lasted **4 weeks or more**. These can be symptoms that continued since their illness, symptoms that got better over time and then returned, or new symptoms. Do not choose symptoms that your child had before their COVID-19 illness.

Energy Level, Memory, and Balance Symptoms

	Did child have this symptom for 4 weeks or longer?	When did this symptom start?	Are they still experiencing this symptom?	If they are no longer experiencing this symptom, when did it get better?
Symptoms that get worse after even mild physical activity or mental effort	○ Yes<br>○ No	○ When they got COVID-19<br>○ Less than 1 month after<br>○ 1-2 months after<br>○ More than 2 months after<br>○ Not sure	○ Yes<br>○ No	○ Within the past few days<br>○ 1-2 weeks ago<br>○ 3-4 weeks ago<br>○ Between 1 and 2 months ago<br>○ More than 2 months ago<br>○ Not sure

	Did child have this symptom for 4 weeks or longer?	When did this symptom start?	Are they still experiencing this symptom?	If they are no longer experiencing this symptom, when did it get better?
Problems sleeping	○ Yes<br>○ No	○ When they got COVID-19<br>○ Less than 1 month after<br>○ 1-2 months after<br>○ More than 2 months after<br>○ Not sure	○ Yes<br>○ No	○ Within the past few days<br>○ 1-2 weeks ago<br>○ 3-4 weeks ago<br>○ Between 1 and 2 months ago<br>○ More than 2 months ago<br>○ Not sure
Problems speaking or communicating	○ Yes<br>○ No	○ When they got COVID-19<br>○ Less than 1 month after<br>○ 1-2 months after<br>○ More than 2 months after<br>○ Not sure	○ Yes<br>○ No	○ Within the past few days<br>○ 1-2 weeks ago<br>○ 3-4 weeks ago<br>○ Between 1 and 2 months ago<br>○ More than 2 months ago<br>○ Not sure
Difficulty thinking clearly or concentrating, forgetfulness, memory loss, or 'brain fog'	○ Yes<br>○ No	○ When they got COVID-19<br>○ Less than 1 month after<br>○ 1-2 months after<br>○ More than 2 months after<br>○ Not sure	○ Yes<br>○ No	○ Within the past few days<br>○ 1-2 weeks ago<br>○ 3-4 weeks ago<br>○ Between 1 and 2 months ago<br>○ More than 2 months ago<br>○ Not sure
Problems with balance or movement	○ Yes<br>○ No	○ When they got COVID-19<br>○ Less than 1 month after<br>○ 1-2 months after<br>○ More than 2 months after<br>○ Not sure	○ Yes<br>○ No	○ Within the past few days<br>○ 1-2 weeks ago<br>○ 3-4 weeks ago<br>○ Between 1 and 2 months ago<br>○ More than 2 months ago<br>○ Not sure

	Did child have this symptom for 4 weeks or longer?	When did this symptom start?	Are they still experiencing this symptom?	If they are no longer experiencing this symptom, when did it get better?
Dizziness, lightheadedness, or fainting	○ Yes<br>○ No	○ When they got COVID-19<br>○ Less than 1 month after<br>○ 1-2 months after<br>○ More than 2 months after<br>○ Not sure	○ Yes<br>○ No	○ Within the past few days<br>○ 1-2 weeks ago<br>○ 3-4 weeks ago<br>○ Between 1 and 2 months ago<br>○ More than 2 months ago<br>○ Not sure
My child did not experience any of these symptoms.	○ My child did not experience any of these symptoms.			

11. Again, thinking about the time between your child's most recent COVID-19 illness and today, choose the symptoms your child had that lasted **4 weeks or more**. These can be symptoms that continued since their illness, symptoms that got better over time and then returned, or new symptoms. Do not choose symptoms that your child had before their COVID-19 illness.

Digestive, Ear, and Eye Symptoms

	Did child have this symptom for 4 weeks or longer?	When did this symptom start?	Are they still experiencing this symptom?	If they are no longer experiencing this symptom, when did it get better?
Appetite changes (ex: eating more than normal, eating less than normal)	○ Yes<br>○ No	○ When they got COVID-19<br>○ Less than 1 month after<br>○ 1-2 months after<br>○ More than 2 months after<br>○ Not sure	○ Yes<br>○ No	○ Within the past few days<br>○ 1-2 weeks ago<br>○ 3-4 weeks ago<br>○ Between 1 and 2 months ago<br>○ More than 2 months ago<br>○ Not sure

	Did child have this symptom for 4 weeks or longer?	When did this symptom start?	Are they still experiencing this symptom?	If they are no longer experiencing this symptom, when did it get better?
Problems swallowing or chewing	○ Yes<br>○ No	○ When they got COVID-19<br>○ Less than 1 month after<br>○ 1-2 months after<br>○ More than 2 months after<br>○ Not sure	○ Yes<br>○ No	○ Within the past few days<br>○ 1-2 weeks ago<br>○ 3-4 weeks ago<br>○ Between 1 and 2 months ago<br>○ More than 2 months ago<br>○ Not sure
Reflux or heartburn	○ Yes<br>○ No	○ When they got COVID-19<br>○ Less than 1 month after<br>○ 1-2 months after<br>○ More than 2 months after<br>○ Not sure	○ Yes<br>○ No	○ Within the past few days<br>○ 1-2 weeks ago<br>○ 3-4 weeks ago<br>○ Between 1 and 2 months ago<br>○ More than 2 months ago<br>○ Not sure
Constipation	○ Yes<br>○ No	○ When they got COVID-19<br>○ Less than 1 month after<br>○ 1-2 months after<br>○ More than 2 months after<br>○ Not sure	○ Yes<br>○ No	○ Within the past few days<br>○ 1-2 weeks ago<br>○ 3-4 weeks ago<br>○ Between 1 and 2 months ago<br>○ More than 2 months ago<br>○ Not sure
Tingling or numbness in any part of body	○ Yes<br>○ No	○ When they got COVID-19<br>○ Less than 1 month after<br>○ 1-2 months after<br>○ More than 2 months after<br>○ Not sure	○ Yes<br>○ No	○ Within the past few days<br>○ 1-2 weeks ago<br>○ 3-4 weeks ago<br>○ Between 1 and 2 months ago<br>○ More than 2 months ago<br>○ Not sure

	Did child have this symptom for 4 weeks or longer?	When did this symptom start?	Are they still experiencing this symptom?	If they are no longer experiencing this symptom, when did it get better?
Ringing in ears (tinnitus)	○ Yes<br>○ No	○ When they got COVID-19<br>○ Less than 1 month after<br>○ 1-2 months after<br>○ More than 2 months after<br>○ Not sure	○ Yes<br>○ No	○ Within the past few days<br>○ 1-2 weeks ago<br>○ 3-4 weeks ago<br>○ Between 1 and 2 months ago<br>○ More than 2 months ago<br>○ Not sure
Eye symptoms (pink eye, conjunctivitis, red eyes, excessive tearing, etc.)	○ Yes<br>○ No	○ When they got COVID-19<br>○ Less than 1 month after<br>○ 1-2 months after<br>○ More than 2 months after<br>○ Not sure	○ Yes<br>○ No	○ Within the past few days<br>○ 1-2 weeks ago<br>○ 3-4 weeks ago<br>○ Between 1 and 2 months ago<br>○ More than 2 months ago<br>○ Not sure
My child did not experience any of these symptoms.	○ My child did not experience any of these symptoms.			

12. Again, thinking about the time between your child's most recent COVID-19 illness and today, choose the symptoms your child had that lasted **4 weeks or more**. These can be symptoms that continued since their illness, symptoms that got better over time and then returned, or new symptoms. Do not choose symptoms that your child had before their COVID-19 illness.

Heart Symptoms

	Did child have this symptom for 4 weeks or longer?	When did this symptom start?	Are they still experiencing this symptom?	If they are no longer experiencing this symptom, when did it get better?
Heart racing or pounding (palpitations)	○ Yes<br>○ No	○ When they got COVID-19<br>○ Less than 1 month after<br>○ 1-2 months after<br>○ More than 2 months after<br>○ Not sure	○ Yes<br>○ No	○ Within the past few days<br>○ 1-2 weeks ago<br>○ 3-4 weeks ago<br>○ Between 1 and 2 months ago<br>○ More than 2 months ago<br>○ Not sure

	Did child have this symptom for 4 weeks or longer?	When did this symptom start?	Are they still experiencing this symptom?	If they are no longer experiencing this symptom, when did it get better?
Irregular Heartbeat (arrythmia)	○ Yes<br>○ No	○ When they got COVID-19<br>○ Less than 1 month after<br>○ 1-2 months after<br>○ More than 2 months after<br>○ Not sure	○ Yes<br>○ No	○ Within the past few days<br>○ 1-2 weeks ago<br>○ 3-4 weeks ago<br>○ Between 1 and 2 months ago<br>○ More than 2 months ago<br>○ Not sure
Faster than normal heart rate	○ Yes<br>○ No	○ When they got COVID-19<br>○ Less than 1 month after<br>○ 1-2 months after<br>○ More than 2 months after<br>○ Not sure	○ Yes<br>○ No	○ Within the past few days<br>○ 1-2 weeks ago<br>○ 3-4 weeks ago<br>○ Between 1 and 2 months ago<br>○ More than 2 months ago<br>○ Not sure
Slower than normal heart rate	○ Yes<br>○ No	○ When they got COVID-19<br>○ Less than 1 month after<br>○ 1-2 months after<br>○ More than 2 months after<br>○ Not sure	○ Yes<br>○ No	○ Within the past few days<br>○ 1-2 weeks ago<br>○ 3-4 weeks ago<br>○ Between 1 and 2 months ago<br>○ More than 2 months ago<br>○ Not sure
New high blood pressure	○ Yes<br>○ No	○ When they got COVID-19<br>○ Less than 1 month after<br>○ 1-2 months after<br>○ More than 2 months after<br>○ Not sure	○ Yes<br>○ No	○ Within the past few days<br>○ 1-2 weeks ago<br>○ 3-4 weeks ago<br>○ Between 1 and 2 months ago<br>○ More than 2 months ago<br>○ Not sure

	Did child have this symptom for 4 weeks or longer?	When did this symptom start?	Are they still experiencing this symptom?	If they are no longer experiencing this symptom, when did it get better?
Chest pain or pressure	○ Yes<br>○ No	○ When they got COVID-19<br>○ Less than 1 month after<br>○ 1-2 months after<br>○ More than 2 months after<br>○ Not sure	○ Yes<br>○ No	○ Within the past few days<br>○ 1-2 weeks ago<br>○ 3-4 weeks ago<br>○ Between 1 and 2 months ago<br>○ More than 2 months ago<br>○ Not sure
My child did not experience any of these symptoms.	○ My child did not experience any of these symptoms.			

13. Again, thinking about the time between your child's most recent COVID-19 illness and today, choose the symptoms your child had that lasted **4 weeks or more**. These can be symptoms that continued since their illness, symptoms that got better over time and then returned, or new symptoms. Do not choose symptoms that your child had before their COVID-19 illness.

Other Symptoms

	Did child have this symptom for 4 weeks or longer?	When did this symptom start?	Are they still experiencing this symptom?	If they are no longer experiencing this symptom, when did it get better?
Bruising/ bleeding easily	○ Yes<br>○ No	○ When they got COVID-19<br>○ Less than 1 month after<br>○ 1-2 months after<br>○ More than 2 months after<br>○ Not sure	○ Yes<br>○ No	○ Within the past few days<br>○ 1-2 weeks ago<br>○ 3-4 weeks ago<br>○ Between 1 and 2 months ago<br>○ More than 2 months ago<br>○ Not sure

	Did child have this symptom for 4 weeks or longer?	When did this symptom start?	Are they still experiencing this symptom?	If they are no longer experiencing this symptom, when did it get better?
Changes in their period (menstrual cycle). For example: irregular periods, shorter or longer cycles	○ Yes<br>○ No<br>○ Not applicable	○ When they got COVID-19<br>○ Less than 1 month after<br>○ 1-2 months after<br>○ More than 2 months after<br>○ Not sure	○ Yes<br>○ No	○ Within the past few days<br>○ 1-2 weeks ago<br>○ 3-4 weeks ago<br>○ Between 1 and 2 months ago<br>○ More than 2 months ago<br>○ Not sure
Hair loss	○ Yes<br>○ No	○ When they got COVID-19<br>○ Less than 1 month after<br>○ 1-2 months after<br>○ More than 2 months after<br>○ Not sure	○ Yes<br>○ No	○ Within the past few days<br>○ 1-2 weeks ago<br>○ 3-4 weeks ago<br>○ Between 1 and 2 months ago<br>○ More than 2 months ago<br>○ Not sure
Joint swelling	○ Yes<br>○ No	○ When they got COVID-19<br>○ Less than 1 month after<br>○ 1-2 months after<br>○ More than 2 months after<br>○ Not sure	○ Yes<br>○ No	○ Within the past few days<br>○ 1-2 weeks ago<br>○ 3-4 weeks ago<br>○ Between 1 and 2 months ago<br>○ More than 2 months ago<br>○ Not sure
Joint pain	○ Yes<br>○ No	○ When they got COVID-19<br>○ Less than 1 month after<br>○ 1-2 months after<br>○ More than 2 months after<br>○ Not sure	○ Yes<br>○ No	○ Within the past few days<br>○ 1-2 weeks ago<br>○ 3-4 weeks ago<br>○ Between 1 and 2 months ago<br>○ More than 2 months ago<br>○ Not sure

	Did child have this symptom for 4 weeks or longer?	When did this symptom start?	Are they still experiencing this symptom?	If they are no longer experiencing this symptom, when did it get better?
Skin changes or rash (COVID toes, hive-like rashes, discoloration, etc.)	○ Yes<br>○ No	○ When they got COVID-19<br>○ Less than 1 month after<br>○ 1-2 months after<br>○ More than 2 months after<br>○ Not sure	○ Yes<br>○ No	○ Within the past few days<br>○ 1-2 weeks ago<br>○ 3-4 weeks ago<br>○ Between 1 and 2 months ago<br>○ More than 2 months ago<br>○ Not sure
Weight loss when they weren't trying to lose weight	○ Yes<br>○ No	○ When they got COVID-19<br>○ Less than 1 month after<br>○ 1-2 months after<br>○ More than 2 months after<br>○ Not sure	○ Yes<br>○ No	○ Within the past few days<br>○ 1-2 weeks ago<br>○ 3-4 weeks ago<br>○ Between 1 and 2 months ago<br>○ More than 2 months ago<br>○ Not sure
My child did not experience any of these symptoms.	○ My child did not experience any of these symptoms.			

14. Again, thinking about the time between your child's most recent COVID-19 illness and today, choose the symptoms your child had that lasted **4 weeks or more**. These can be symptoms that continued since their illness, symptoms that got better over time and then returned, or new symptoms. Do not choose symptoms that your child had before their COVID-19 illness.

Well-Being and Mood Symptoms

	Did child have this symptom for 4 weeks or longer?	When did this symptom start?	Are they still experiencing this symptom?	If they are no longer experiencing this symptom, when did it get better?
Feeling anxious, restless, or on edge; unable to stop worrying; trouble relaxing; irritability	○ Yes<br>○ No	○ When they got COVID-19<br>○ Less than 1 month after<br>○ 1-2 months after<br>○ More than 2 months after<br>○ Not sure	○ Yes<br>○ No	○ Within the past few days<br>○ 1-2 weeks ago<br>○ 3-4 weeks ago<br>○ Between 1 and 2 months ago<br>○ More than 2 months ago<br>○ Not sure

	Did child have this symptom for 4 weeks or longer?	When did this symptom start?	Are they still experiencing this symptom?	If they are no longer experiencing this symptom, when did it get better?
Feelings of sadness, hopelessness, or loss of interest or pleasure in activities	○ Yes<br>○ No	○ When they got COVID-19<br>○ Less than 1 month after<br>○ 1-2 months after<br>○ More than 2 months after<br>○ Not sure	○ Yes<br>○ No	○ Within the past few days<br>○ 1-2 weeks ago<br>○ 3-4 weeks ago<br>○ Between 1 and 2 months ago<br>○ More than 2 months ago<br>○ Not sure
Having nightmares, unwanted memories of trauma	○ Yes<br>○ No	○ When they got COVID-19<br>○ Less than 1 month after<br>○ 1-2 months after<br>○ More than 2 months after<br>○ Not sure	○ Yes<br>○ No	○ Within the past few days<br>○ 1-2 weeks ago<br>○ 3-4 weeks ago<br>○ Between 1 and 2 months ago<br>○ More than 2 months ago<br>○ Not sure
Mood swings (sudden or frequent changes in their emotions or mood)	○ Yes<br>○ No	○ When they got COVID-19<br>○ Less than 1 month after<br>○ 1-2 months after<br>○ More than 2 months after<br>○ Not sure	○ Yes<br>○ No	○ Within the past few days<br>○ 1-2 weeks ago<br>○ 3-4 weeks ago<br>○ Between 1 and 2 months ago<br>○ More than 2 months ago<br>○ Not sure
My child did not experience any of these symptoms.	○ My child did not experience any of these symptoms.			

15. Again, thinking about the time between your child's most recent COVID-19 illness and today, **did your child have any other symptoms that lasted 4 weeks or more**? These can include ongoing symptoms from the time of COVID-19 illness, symptoms that got better and then returned, or new symptoms. Do not include symptoms that your child usually had before their COVID-19 illness.

	Did child have this symptom for 4 weeks or longer?	When did this symptom start?	Are they still experiencing this symptom?	If they are no longer experiencing this symptom, when did it get better?
My child did not experience any other symptoms	○ My child did not experience any other symptoms.			
Other, please specify: ______________ ______________ ______________ ________	○ Yes<br>○ No	○ When they got COVID-19<br>○ Less than 1 month after<br>○ 1-2 months after<br>○ More than 2 months after<br>○ Not sure	○ Yes<br>○ No	○ Within the past few days<br>○ 1-2 weeks ago<br>○ 3-4 weeks ago<br>○ Between 1 and 2 months ago<br>○ More than 2 months ago<br>○ Not sure
Other, please specify: ______________ ______________ ______________ ________	○ Yes<br>○ No	○ When they got COVID-19<br>○ Less than 1 month after<br>○ 1-2 months after<br>○ More than 2 months after<br>○ Not sure	○ Yes<br>○ No	○ Within the past few days<br>○ 1-2 weeks ago<br>○ 3-4 weeks ago<br>○ Between 1 and 2 months ago<br>○ More than 2 months ago<br>○ Not sure
Other, please specify: ______________ ______________ ______________ ________	○ Yes<br>○ No	○ When they got COVID-19<br>○ Less than 1 month after<br>○ 1-2 months after<br>○ More than 2 months after<br>○ Not sure	○ Yes<br>○ No	○ Within the past few days<br>○ 1-2 weeks ago<br>○ 3-4 weeks ago<br>○ Between 1 and 2 months ago<br>○ More than 2 months ago<br>○ Not sure

16. Are there any other symptoms you haven't already mentioned that have gotten WORSE since your child's most recent COVID-19 illness?
   ○ Yes
   ○ No → **Skip to #18**

17. Please describe these symptoms.

If no symptoms were marked or mentioned in questions #9–#17, → skip to #21.

18. Did your child see a doctor, nurse, or other health professional or visit a hospital or other health facility (such as an urgent care, clinic, doctor's office, or health department) for any of these symptom(s) you marked in questions #9-#17?
   ○ Yes
   ○ No
   ○ Prefer not to answer

19. How much difficulty did/do these symptoms cause with your child's day-to-day life? This can be school, work, hobbies, or other regular activities?
   ○ No difficulty
   ○ Mild difficulty
   ○ Moderate difficulty
   ○ Severe difficulty
   ○ Extreme difficulty, or unable to do these activities

20. Have you been told by a health care provider that your child has Long COVID, Post-COVID Conditions, or Post-Acute Sequelae of COVID-19 (PASC)?
   ○ Yes
   ○ No
   ○ Not sure
   ○ Prefer not to answer

Past Medical History

21. Below are some common health conditions. Have you ever been told by a doctor or health care professional that your child has or had any of these conditions? If so, did these conditions first occur before or after their most recent COVID-19 illness? **Mark the circles that apply for each condition.**

	Have you ever been told that they had this condition? ***Mark all that apply***	Did this condition first occur before or after their first positive COVID-19 test result/diagnosis?	Do they still have this condition?
Chronic lung disease, such as asthma or COPD (chronic obstructive pulmonary disease)	○ Yes<br>○ No	○ Before<br>○ After	○ Yes<br>○ No
A mental health disorder, such as anxiety or depression	○ Yes<br>○ No	○ Before<br>○ After	○ Yes<br>○ No
An immune system disorder, such as Parkinson's, systemic lupus, multiple sclerosis, chronic fatigue syndrome, rheumatoid arthritis, HIV, having been a recipient of an organ or bone marrow transplant, being on immunosuppressive medication, missing your spleen, or severe combined immunoglobulin deficiency (SCID)	○ Yes<br>○ No	○ Before<br>○ After	
Cancer, except skin cancer	○ Yes<br>○ No	○ Before<br>○ After	○ Yes<br>○ No
Chronic kidney disease, such as chronic renal failure, end-stage renal failure or renal failure requiring dialysis	○ Yes<br>○ No	○ Before<br>○ After	
Chronic liver disease, such as fatty liver, steatohepatitis, alcoholic liver disease or chronic hepatitis C	○ Yes<br>○ No	○ Before<br>○ After	
Chronic headaches or migraines	○ Yes<br>○ No	○ Before<br>○ After	○ Yes<br>○ No
Heart disease, other cardiovascular disease, or heart attack	○ Yes<br>○ No	○ Before<br>○ After	
High blood pressure	○ Yes<br>○ No	○ Before<br>○ After	○ Yes<br>○ No

	Have you ever been told that they had this condition? ***Mark all that apply***	Did this condition first occur before or after their first positive COVID-19 test result/diagnosis?	Do they still have this condition?
High cholesterol	○ Yes<br>○ No	○ Before<br>○ After	○ Yes<br>○ No
Diabetes, **Check type:**<br>☐ Type 1 ☐ Type 2<br>☐ Gestational ☐ Not sure	○ Yes<br>○ No	○ Before<br>○ After	○ Yes<br>○ No
Chronic pain	○ Yes<br>○ No	○ Before<br>○ After	○ Yes<br>○ No
Stroke	○ Yes<br>○ No	○ Before<br>○ After	
Obesity	○ Yes<br>○ No	○ Before<br>○ After	○ Yes<br>○ No
Other, please specify:<br>_______________	○ Yes<br>○ No	○ Before<br>○ After	○ Yes<br>○ No
Other, please specify:<br>_______________	○ Yes<br>○ No	○ Before<br>○ After	○ Yes<br>○ No
None of these	○ None of these		

22. Does your child have any other health conditions or concerns that have been made <u>worse</u> by COVID-19?
   - ○ Yes, please specify: _______________
   - ○ No

23. What is your child's height?

   ☐☐ Feet ☐☐ Inches

   - ○ Don't know
   - ○ Prefer not to answer

24. What is your child's weight?

   ☐☐☐ Pounds

   - ○ Don't know
   - ○ Prefer not to answer

COVID-19 Vaccination

The following questions are about COVID-19 vaccinations your child may have received. If you have your child's vaccination card or records, that may help you answer these questions. If you do not have your child's card or vaccination record or you cannot remember please use your best guess.

25. Has your child ever received a COVID-19 vaccine?
   - ○ Yes
   - ○ No → **Skip to #31**
   - ○ I don't know → **Skip to #31**
   - ○ Prefer not to answer → **Skip to #31**

26. Did your child receive a COVID-19 vaccine **before** their COVID-19 illness you entered for question #1?
   - ○ Yes
   - ○ No → **Skip to #28**
   - ○ I don't know → **Skip to #28**
   - ○ Prefer not to answer → **Skip to #28**

27. When was your child's most recent COVID-19 vaccine *before* their COVID-19 illness you entered for question #1?

Month		Day		Year			
				2	0		

   - ○ I don't know
   - ○ Prefer not to answer

28. Did your child receive a COVID-19 vaccination **after** their COVID-19 illness you entered for question #1?
   - ○ Yes
   - ○ No → **Skip to #30**
   - ○ I don't know → **Skip to #30**
   - ○ Prefer not to answer → **Skip to #30**

29. When was your child's most recent COVID-19 vaccination?

Month		Day		Year			
				2	0		

   - ○ I don't know
   - ○ Prefer not to answer

30. How many total COVID-19 shots has your child had?

Total COVID-19 shots

○ I don't know
○ Prefer not to answer

General Wellbeing Assessment

31. In general, would you say your child's health is:
○ Excellent
○ Very good
○ Good
○ Fair
○ Poor

32. In general, would you say your child's quality of life is:
○ Excellent
○ Very good
○ Good
○ Fair
○ Poor

33. In general, how would you rate your child's physical health?
○ Excellent
○ Very good
○ Good
○ Fair
○ Poor

34. In general, how would you rate your child's mental health, including their mood and ability to think?
○ Excellent
○ Very good
○ Good
○ Fair
○ Poor

35. How often does your child feel really sad?
○ Never
○ Rarely
○ Sometimes
○ Often
○ Always

36. How often does your child have fun with friends?
○ Always
○ Often
○ Sometimes
○ Rarely
○ Never

37. How often does your child feel that their parent/caregiver listens to their ideas?
   - ○ Always
   - ○ Often
   - ○ Sometimes
   - ○ Rarely
   - ○ Never

38. In the past 7 days, my child got tired easily.
   - ○ Never
   - ○ Rarely
   - ○ Sometimes
   - ○ Often
   - ○ Almost always

39. In the past 7 days, my child had trouble sleeping when they had pain.
   - ○ Never
   - ○ Almost Never
   - ○ Sometimes
   - ○ Often
   - ○ Almost Always

40. In the past 7 days, how would you rate your child's pain on average?

No pain										Worst pain imaginable
0	1	2	3	4	5	6	7	8	9	10
○	○	○	○	○	○	○	○	○	○	○

Mental Wellbeing

Below are some statements about feelings and thoughts. **Please mark the answer that best describes your child's experience of each statement over the last 14 days**.

	None of the time	Rarely	Some of the time	Often	All of the time
41. They've been feeling optimistic about the future	○	○	○	○	○
42. They've been feeling useful	○	○	○	○	○
43. They've been feeling relaxed	○	○	○	○	○
44. They've been feeling interested in other people	○	○	○	○	○
45. They've had energy to spare	○	○	○	○	○
46. They've been dealing with problems well	○	○	○	○	○
47. They've been thinking clearly	○	○	○	○	○
48. They've been feeling good about themself	○	○	○	○	○

	None of the time	Rarely	Some of the time	Often	All of the time
49. They've been feeling close to other people	○	○	○	○	○
50. They've been feeling confident	○	○	○	○	○
51. They've been able to make up their own mind about things	○	○	○	○	○
52. They've been feeling loved	○	○	○	○	○
53. They've been interested in new things	○	○	○	○	○
54. They've been feeling cheerful	○	○	○	○	○

Fatigue Assessment

Please respond to each question or statement by **marking one answer for each item.**
In the past 7 days:

	Never	Almost Never	Somewhat	Often	Almost Always
55. Being tired made it hard for my child to keep up with their schoolwork	○	○	○	○	○
56. Being tired made it hard for my child to play or go out with their friends as much as they'd like	○	○	○	○	○
57. My child felt weak	○	○	○	○	○
58. My child got tired easily	○	○	○	○	○
59. My child had trouble finishing things because they were too tired	○	○	○	○	○
60. My child had trouble starting things because they were too tired	○	○	○	○	○
61. My child was so tired it was hard for them to pay attention	○	○	○	○	○
62. My child was too tired to do sports or exercise	○	○	○	○	○
63. My child was too tired to do things outside	○	○	○	○	○
64. My child was too tired to enjoy the things they like to do	○	○	○	○	○

Cognitive Function Assessment

In the past 7 days:

	Never	Rarely (Once)	Sometimes (Two or three times)	Often (About once a day)	Very often (Several times a day)
65. My child's thinking has been slow...	○	○	○	○	○
66. It has seemed like my child's brain was not working as well as usual...	○	○	○	○	○
67. My child has had to work harder than usual to keep track of what they were doing...	○	○	○	○	○
68. My child had trouble shifting back and forth between different activities that require thinking...	○	○	○	○	○
69. My child has had trouble concentrating...	○	○	○	○	○
70. My child has had to work really hard to pay attention or they would make a mistake...	○	○	○	○	○

Impact on Work/School Assessment

71. In the past 7 days: Did your child take time off school due to illness? This includes if they are homeschooled.
○ Yes
○ No → **Skip to #73**
○ Not applicable – They don't go to school → **Skip to #73**
○ Prefer not to answer → **Skip to #73**

72. How many days did your child take off in the past 7 days?

☐ Days

73. In the past 7 days, did someone else take time off work or school to care for your child due to their illness?
○ Yes
○ No → **Skip to #75**
○ Prefer not to answer → **Skip to #75**

74. In the past 7 days, how many days did someone else take time off to care for your child?

☐ Days

Demographics

75. What sex was your child assigned at birth?
    - ○ Male
    - ○ Female
    - ○ Prefer not to answer

76. Does your child currently describe themself as:
    - ○ Male
    - ○ Female
    - ○ Transgender female
    - ○ Transgender male
    - ○ They use a different term, please specify:______________________________
    - ○ Prefer not to answer

77. What is your child's date of birth?

Month Day Year

78. What is your child's ethnicity?
    - ○ Hispanic
    - ○ Non-Hispanic
    - ○ Prefer not to answer

79. What is your child's race? **Select all that apply.**
    - ○ Asian or Asian American
    - ○ Black or African American
    - ○ Native Hawaiian or other Pacific Islander
    - ○ American Indian or Alaska Native
    - ○ White or Caucasian
    - ○ Other race, please specify:______________________________
    - ○ Prefer not to answer

80. How many people currently live in your child's household, including your child?

    [ ][ ] Total number people living in household

    - ○ My child lives in a group setting such as a group foster home → **Skip to #82**
    - ○ Prefer not to answer → **Skip to #82**

81. How many children under 18 years of age currently live in your household (including your child)?

    [ ][ ] Number of children under 18 living in household

82. What is the highest degree or level of school that your child has completed?
- ○ Never attended school or only attended kindergarten
- ○ Grades 1- 6 (or homeschool equivalent)
- ○ Grades 7-8 (or homeschool equivalent)
- ○ Grades 9-12 (or homeschool equivalent)
- ○ High school graduate or GED
- ○ College, 1-3 years, or some college, associate's degree, or technical school

83. What kind(s) of health insurance or health care coverage does your child have? **Mark all that apply.**
- ○ Medicare (Including Medicare Advantage plans)
- ○ Medicaid (Names of these plans may differ by state, for example Medi Cal in California)
- ○ Private (either employer-provided or an individually purchased plan, including plans purchased under the Affordable Care Act and COBRA, and temporary health insurance)
- ○ Military insurance, such as Tricare
- ○ Children's Health Insurance Program, or CHIP
- ○ Other, please specify: ____
- ○ No health care coverage of any type
- ○ Not sure
- ○ Prefer not to answer

84. Does your child qualify for free or low-cost breakfast or lunch at their school?
- ○ Yes
- ○ No
- ○ I don't know
- ○ My child is home-schooled

85. Did you receive assistance from someone (for example, a family member or caregiver) to complete this survey?
- ○ Yes
- ○ No → **Skip to #86**

85a. Who assisted you in completing this survey?

86. Has any member of your household taken a survey as part of this project?
- ○ Yes
- ○ No
- ○ I'm not sure
